# Regulation of GH and GH Signaling by Nutrients

**DOI:** 10.3390/cells10061376

**Published:** 2021-06-02

**Authors:** Marina Caputo, Stella Pigni, Emanuela Agosti, Tommaso Daffara, Alice Ferrero, Nicoletta Filigheddu, Flavia Prodam

**Affiliations:** 1 SCDU of Endocrinology, University Hospital Maggiore della Carità, 28100 Novara, Italy; marina.caputo@uniupo.it (M.C.); 20007328@studenti.uniupo.it (S.P.); tdaffara@gmail.com (T.D.); alli.ferrero@gmail.com (A.F.); 2 Department of Health Sciences, Università del Piemonte Orientale, 28100 Novara, Italy; emanuela.agosti@med.uniupo.it; 3 Department of Translational Medicine, Università del Piemonte Orientale, 28100 Novara, Italy; nicoletta.filigheddu@med.uniupo.it

**Keywords:** GH, IGF-I, regulation, nutrient, vitamin, mineral, food, fasting, feeding, diet

## Abstract

Growth hormone (GH) and insulin-like growth factor-1 (IGF-I) are pleiotropic hormones with important roles in lifespan. They promote growth, anabolic actions, and body maintenance, and in conditions of energy deprivation, favor catabolic feedback mechanisms switching from carbohydrate oxidation to lipolysis, with the aim to preserve protein storages and survival. IGF-I/insulin signaling was also the first one identified in the regulation of lifespan in relation to the nutrient-sensing. Indeed, nutrients are crucial modifiers of the GH/IGF-I axis, and these hormones also regulate the complex orchestration of utilization of nutrients in cell and tissues. The aim of this review is to summarize current knowledge on the reciprocal feedback among the GH/IGF-I axis, macro and micronutrients, and dietary regimens, including caloric restriction. Expanding the depth of information on this topic could open perspectives in nutrition management, prevention, and treatment of GH/IGF-I deficiency or excess during life.

## 1. Introduction

Growth hormone (GH) and insulin-like growth factor-1 (IGF-I) are pleiotropic hormones with important roles in lifespan. They regulate growth, maintenance of lean and bone mass, as well as cellular differentiation, function, and survival, acting on several mechanisms including mitochondria homeostasis, a keystone for metabolic processing of carbohydrates, fats, and amino acids (AA) [1,2,3].

Because the GH/IGF-I axis promotes growth, direct and indirect anabolic actions are obvious effects. AA uptake, RNA, and protein synthesis are all promoted by the two hormones with the involvements of other anabolic stimuli, like that of insulin, when energy conditions are advantageous. Diversely, in the condition of energy deprivation, GH/IGF-I favors catabolic feedback characterized by a switch from carbohydrate oxidation to lipolysis, with the aim to preserve protein storages [1,2]. 

GH and IGF-I secretion progressively decline with age. Growing evidence suggests this phenomenon as a protective adaptation to altered functions and diseases typical of aging, but also overnutrition [2,4,5]. However, the GH/IGF-I axis is strongly influenced by the nutritional state in any phase of life. This review aims to briefly summarize the mechanisms related to the regulation of GH and IGF-I by nutrients. We highlighted how both macronutrients and micronutrients have a complex orchestration on the axis, suggesting that balanced nutrition, which prevents nutrient deficiencies and overloads, is essential for the GH/IGF-I homeostasis. Furthermore, we focused on how diet regimens could modulate the somatotroph axis.

## 2. GH and IGF-I Structure, Regulation, and Signaling

GH is a peptide hormone characterized by several molecular isoforms. Among the causes of GH heterogenicity, gene duplication, mRNA splicing, post-translational modifications, and GH metabolism can be cited. Specifically, five genes are located at the genetic locus encoding for GH on human chromosome 17q24.2: the GH genes GH1 and GH2, and the human chorionic somatomammotropin genes CSH1, CSH2, and CSP1 [6]. The predominant GH variant is a 22 kDa single-chain protein with two disulfide bridges that derives from the processing of the long pre-GH transcript encoded by the GH1 gene, which also yields a 20 kDa variant by alternative splicing. While the GH1 gene is expressed in pituitary somatotrophs, the GH2 gene is expressed in the placenta and encodes for a 22 kDa GH variant that has a role in fetal development [7]. In addition to these variants, the heterogeneous mixture of GH isoforms in the blood is also dependent on a great variety of post-translational modifications, such as N(α)-acylation, glycosylation, and deamidation [7]. GH is secreted by the somatotroph cells of the anterior pituitary in a pulsatile manner characterized by secretory episodes separated by intervals of relative secretory quiescence with undetectable GH levels. Particularly, two-thirds of GH secretion occurs during the night, with 70% of GH released with the first episode of slow-wave sleep [8]. Two hypothalamic peptides function as primary regulators of somatotroph secretion: GH-releasing hormone (GHRH) and somatostatin, which stimulate and inhibit GH synthesis and release, respectively [9]. GHRH, released from neurosecretory nerve terminals of arcuate neurons, interacts with the G protein-coupled receptor GHRHR on the somatotroph cells of the adenohypophysis, promoting GH release through cAMP signaling activation or acting on GH-containing secretory vesicles [10]. Somatostatin is produced by neuroendocrine neurons of the ventromedial nucleus of the hypothalamus and inhibits GHRH-induced GH secretion upon binding to G protein-coupled receptors (sst1, sst2, sst3, and sst5) [11]. Several factors are responsible for the somatostatin-mediated regulation of GH levels, including serum levels of GH/IGF-I and glucose, as well as exercise and immobilization [12]. To complicate this scenario, a complex network of neurotransmitters and neuropeptides further regulates the secretion of GHRH and somatostatin at other hypothalamic and supra-hypothalamic levels [13].

Furthermore, GH secretory bursts vary in frequency and amplitude according to age, gender, pubertal status, menstrual cycle phase, sleep, exercise, nutritional status, and body composition [9]. Most of these factors likely influence somatotroph secretion by affecting CNS neurotransmitters and/or other hormones’ concentrations (IGF-I, insulin, ghrelin, gonadal hormones, glucocorticoids) and circulating metabolic fuel levels [14,15,16]. Finally, several macro- and micronutrients potentiate or inhibit GH secretion, and the following sections will detail the mechanisms involved.

The GH intracellular signaling cascade begins with the binding to a dimeric cell surface receptor, called the GH receptor (GHR). GHR is a member of the class I cytokines receptor family highly expressed in liver, muscle, kidney, heart, and skin epidermis and characterized by the lack of intrinsic protein kinase activity [17,18]. Upon GH binding, GHR recruits the non-receptor protein tyrosine kinase JAK2, which phosphorylates several tyrosine residues on the intracellular domain of the receptor, facilitating the recruitment of other signaling molecules [19]. 

The primary signaling pathways activated by GH are the JAK-STAT (signal transducer and activator of transcription) pathway, mitogen-activated protein kinase (MAPK), and phosphatidylinositol 3-kinase/AKT/mechanistic target of rapamycin (PI3K/AKT/mTOR) pathways [20]. The diversity of the signaling pathways activated by GHR can explain, at least in part, the different effects that GH exerts on various tissues.

Many, but not all, of the effects of GH are mediated by IGF-I. IGF-I is a 7.6 kDa protein, a member of a family of insulin-related peptides primarily produced by the liver under the direct influence of GH, although it is now recognized that most of the tissues and cells in the body produce IGF-I and express IGF-I receptor (IGF-IR), as well as GHR [21]. Both IGF-I and GH itself exert a negative feedback on GH secretion at the pituitary level. They work together to promote somatic growth, and they both modulate metabolic functions with synergistic as well as opposing actions [1,21]. As suggested by the name, IGF-I and insulin show a high degree of structural and functional homology, and this holds for their respective receptors too, leading to important crossover effects on growth and metabolism (i.e., anabolic, lipotropic, and hypoglycemic actions) [21,22,23]. Similarly to GH, upon the ligand binding, IGF-IR undergoes structural changes that result in the autophosphorylation of many tyrosine residues in the b-subunit of the receptor and the recruitment of docking proteins such as IRS1, IRS2, and Shc. In particular, the PH domain of IRS recruits major insulin-like signaling molecules to the membrane, leading to the activation of PI3K/AKT/mTOR pathway, a well-known nutrient and growth factor sensor that regulates several processes such as cell growth, proliferation, and survival [24]. Moreover, cell growth and differentiation are controlled by the MAPK pathway, activated by Shc [25]. The ability of IGF-I to bind to and activate IGF-IR is regulated by a family of six binding proteins (IGFBP) that act as transporters for IGF-I in the circulation, reducing the amount of free IGF-I and increasing its half-life. The 80% of IGF-I that is complexed into a tertiary complex with IGFBP-3 and a glycoprotein called acid labile subunit (ALS) creates a long-lasting reservoir of IGF-I in the circulation [26,27]. IGFBP-3 is mainly produced in the liver in a GH-regulated manner that ensures an orchestrated increase of both IGF-I and its binding proteins [28]. The liver is also responsible for the synthesis of IGFBP-1, the expression and secretion of which are highly regulated by catabolic factors and hormones. Indeed, starvation, hypoxia, glucocorticoids, and stress induce IGFBP-1, while insulin exerts a negative effect on it [29,30,31].

## 3. GH Secretion during Fasting and Feeding

Nutritional status plays a key role in the regulation of GH secretion, and in turn, GH significantly influence nutrients utilization and metabolism in humans and animals, as briefly mentioned previously.

In humans, the regulatory effects of different nutritional states on GH secretion werefirst investigated in studies mainly conducted in the 1980s and 1990s. Frequent venous sampling to detect pulses in GH concentration and deconvoluted analysis to resolve both GH secretion and clearance rates have been the primary methods used for this purpose [14,32,33]. More recently, studies on the ghrelin-GH axis provided new perspectives into this research field.

### 3.1. Fasting

Fasting has a stimulatory effect on somatotroph secretion in humans [8,34,35], which is consistent with the lipolytic and hyperglycemic properties of GH, promoting endogenous fuel mobilization and utilization in times of food scarcity [36]. Nutritional deprivation increases GH secretory bursts’ frequency and amplitude, without affecting the hormone clearance [8,34]. Prolonged (more than 3 days) fasting leads to peripheral GH resistance, characterized by normal or elevated GH levels coupled with low serum IGF-I concentration, which implies an impairment in IGF-I negative feedback action [14,15,37,38,39].

Accordingly, in disorders characterized by under-nutrition such as anorexia nervosa, both spontaneous and stimulated GH secretion are increased in the setting of low IGF-I levels [40,41,42].

Potential mechanisms contributing to this state of GH resistance in conditions of nutritional deprivation likely include:

- A decline of serum IGF-I due to reduced nutrient intake: besides GH, nutritional status is a key regulator of IGF-I synthesis and secretion, and adequate nutrition is required for the liver response to GH with normal IGF-I production [39,43];
- Down-regulation of the GHR in the liver: studies in animal models of starvation found low IGF-I levels coupled with decreased hepatic GHR mRNA levels and decreased GH binding [44,45]. Low insulin levels, as those seen in the fasting state, may in part mediate GH resistance by reducing surface expression of GHR in the liver [46];
- Post-receptor mechanisms resulting in the inability of GH to stimulate IGF-I production: for example, fibroblast growth factor-21 (FGF-21) and Sirtuin 1 (SIRT1) have been shown to play a role in GH resistance in states of nutritional deprivation acting via STAT5 inhibition [42].

Compared to prolonged fasting, short-term (<3 days) fasting significantly increases GH secretion before any IGF-I reduction [34]. Thus, CNS-mediated mechanisms altering both GHRH and somatostatin secretion have been suggested to be involved in the prompt stimulatory effect of fasting on somatotroph secretion. In fact, based on analyses of the changes in GH inter-burst intervals, fasting has been shown to increase the activity of GHRH-secreting neurons, and prolongs the nadirs of somatostatin secretion [34].

Interestingly, in contrast to normal subjects, both hypopituitary adult patients with GH deficiency (GHD) and acromegalic patients show abnormal GH responses to fasting and food deprivation. Some authors demonstrated that patients with GHD lack the GH response to food deprivation concluding that, theoretically, the assessment of spontaneous GH secretion after a short-term fasting could be useful for the diagnosis of adult GHD [47]. The same group found that in acromegalic patients, the GH/IGF-I axis display some degree of refractoriness to short-term fasting, as, after 36h-of fasting, only control subjects showed increased GH concentrations and significant IGF-I reduction [48]. Further detailed information on changes in GH signaling during fasting are described in the parapragh in Section 6 focused on caloric restriction (CR).

### 3.2. Feeding

In agreement with the previously described mechanisms, refeeding, as well as over-eating, decrease GH secretion. Subjects undergoing refeeding, either after a short-term fasting or in recovery from chronic under-nutrition, show a suppression of previously enhanced GH secretion and serum IGF-I increase, returning to normal or nearly normal levels [40,41,49]. More recently, a study conducted in non-obese healthy men demonstrated that overeating per se can markedly suppress GH secretion before any measurable weight gain. The rapid and consistent GH suppression observed with overfeeding was a consequence of a reduction in GH pulse amplitude, with no change in plasma inter-pulse GH concentration, and was associated with a rapid and sustained elevation in plasma insulin levels [50]. 

Different mechanisms have been proposed to explain the effects of nutritional repletion on GH secretion, including increase in free fatty acid (FFA) concentrations [51,52], alterations in hypothalamic somatostatin and GHRH secretion [14], modification of IGF-I bioavailability by changes in IGFBP concentrations [15,53,54,55], and hyperinsulinemia-mediated inhibition of GH secretion [50,56,57]. 

Such mechanisms have also been associated with obesity-related alterations in the GH/IGF-I axis. In obesity, GH secretion decreases because of a reduction in the amount of GH secreted per burst, while burst frequency is unaffected [58,59]. Moreover, obese patients show severely blunted somatotroph responses to well-known GH stimuli, including fasting [55,60,61,62]. Both spontaneous and stimulated GH secretion normalize after significant weight loss [63,64,65], thus proving that somatotroph insufficiency in obesity is reversible and probably reflects peripheral hormone, metabolic, and possibly neuroendocrine abnormalities in a state of chronic over-nutrition.

### 3.3. Role of the Ghrelin-GH Axis in Fasting and Feeding

Ghrelin is a 28-amino-acid peptide predominantly produced in and secreted by endocrine cells in the stomach, although it is also widely express in other tissues [14]. It was first identified in 1999 as a hormone able to exert a strong GH-releasing effect acting through the GH secretagogue receptor type 1a (GHS-R1a) found in the pituitary gland and hypothalamus [14]. Ghrelin exists in two forms: acylated ghrelin, octanoylated at the Ser3 residue; and unacylated ghrelin lacking this modification. GOAT (ghrelin O-acyltransferase) is the enzyme that specifically octanoylates ghrelin, and this posttranslational modification is essential for binding to GHS-R1a [66]. Acylated ghrelin binds to and activates GHS-R1a, causing the phospholipase C-mediated increase in cytosolic calcium that results in GH release from the somatotrophs cells in the pituitary [67]. In addition, ghrelin can indirectly contribute to GH secretion thanks to its negative activity on IGF-I. Indeed, ghrelin can both remove the inhibitory effects of IGF-I on GH and block the stimulatory effects of IGF-I on somatostatin, allowing the release of GHRH to the portal circulation [68]. Moreover, ghrelin and GHRH colocalize in the hypothalamic arcuate nucleus, where ghrelin can induce GHRH release directly [69]. 

Besides its ability to stimulate GH secretion, ghrelin is a well-known orexigenic hormone, the effects of which are mediated through activation of both hypothalamic and extra-hypothalamic regions involved in the regulation of homeostatic as well as hedonic feeding [70,71,72,73]. Moreover, ghrelin regulates glucose and lipid metabolism, as well as body composition, and has emerging multifaceted roles in cellular/tissue homeostasis [74,75]. Interestingly, ghrelin and asprosin, a recently discovered fasting-induced adipokine with orexigenic and glucogenic actions [76], seem to activate a partially overlapping subset of AgRP neurons within the arcuate nucleus of the hypothalamus, and asprosin-deficiency makes these neurons less responsive to ghrelin-mediated activation [77]. However, the role of asprosin and its crosstalk with other hormones in the energy homeostasis system still requires further investigation. 

Circulating levels of ghrelin increase after fasting and decrease after feeding. With the exception of the neonatal age, they are closely regulated by nutrients, in particular by carbohydrates [78,79,80]. They also negatively correlate with BMI, being lower in obesity and higher in lean people and anorexia nervosa patients [71,80]. Several studies have investigated the relationship between ghrelin and GH under fed or fasting conditions. Although some authors [81,82,83,84] showed a relationship between changes in serum levels of ghrelin and GH, especially in the fasted-state, others failed to demonstrate such a correlation [85,86,87,88]. Nonetheless, ghrelin plays an essential role under starved, fat-depleted conditions allowing GH-mediated maintenance of viable blood glucose levels [79,84,89,90,91]. Further evidence has been provided by the recent discovery of LEAP2 (liver-enriched antimicrobial peptide-2) as an endogenous antagonist of GHS-R1a produced in the liver and small intestine [92]. In mice, LEAP2 administration blocks both ghrelin-induced GH release and food intake in a dose-dependent manner, while LEAP2 neutralization after 24 h of fasting increases GH release. Furthermore, LEAP2 overexpression is associated with severe hypoglycemia and compromises animal survival during chronic CR, likely due to inhibition of the ghrelin-GH axis [92]. LEAP2 circulating levels are decreased by fasting and partially restored by refeeding, while they are higher in obesity and postprandially, positively correlating with BMI. Thus, there is an opposing regulation of LEAP2 and ghrelin in response to both long-term and-short term changes in metabolic and nutritional status [92,93]. This could lead to a reciprocal feedback in which energy restriction results in low LEAP2 concentration and high ghrelin levels, in order to stimulate food intake and avoid hypoglycemic episode. On the other hand, excessive food intake increases LEAP2 with a following decrease in ghrelin to blunt further caloric intake in hyperglycemia [93].

## 4. Regulation of GH and GH Signaling from Macronutrients

### 4.1. Carbohydrates

Carbohydrates are found in a wide variety of natural and processed foods, and are classically divided into simple carbohydrates or sugars (mono- and disaccharides) and complex carbohydrates (oligosaccharides and polysaccharides, also known as starches). The latter are broken down into have sugars that are the major fuel source for metabolism, yielding energy for most of the human cells [94]. Classically, carbohydrates account for at least 45–60% of total dietary energy intake and can be metabolized to provide energy or are stored in muscle and liver as glycogen [95]. The suggested amount of sugars is less than 10–15% of total energy intake to counteract the unfavorable metabolic effects [95,96,97]. The stored reserve is limited, with the muscle being the largest store, although muscular glycogen, opposed to the hepatic one, is not readily available. Considering their limited reserve, de-novo synthesis (gluconeogenesis) of carbohydrates from a source like AA is possible; an excessive intake of carbohydrates is generally oxidized rather than stored, but when the intake is very high, the excess is converted to fatty acids for storage in adipose cells. Indeed, carbohydrates enter in a crossroads of several signal pathways to preserve/use energy and, as consequence, promote growth or maintain human body homeostasis and energy. It is therefore not surprising that the anabolic effects of GH are closed to carbohydrate metabolism.

GH/IGF-I regulation is related to insulin secretion and action, suggesting how this axis is critical for carbohydrate metabolism. In favor of this, some data suggest that GH and insulin actions converge at the postreceptor level [1,98,99] and that GH-mediated IGF-I production requires an adequate portal level of insulin [100]; moreover, insulin modulates the hepatic expression of GHR and influences IGF-I and IGFBP levels, while GH promotes phosphorylation of IRS1 and IRS2 by activation of JAK2. While GH switches metabolism from glucose and protein to lipid [1], IGF-I stimulates glucose uptake and decreases gluconeogenesis and glycogenolysis, improving insulin sensitivity [101]. It was reported that GH administration is followed by insulin resistance and relatively sustained hyperglycemia [1,99,101], probably because of the GH lipolytic effect that inhibits insulin-mediated glucose uptake, especially in the muscle [98] and, partly, in the liver [1]. However, GH is able to induce insulin-resistance before the elevation of circulating FFA, suggesting other mechanisms [1,99] such as the induction of SOCS1 and SOCS3, negative regulators of insulin signaling [102], and the modulation of PI3K activity through increased expression of p85alfa, which binds to IRS and inhibits insulin signaling [103,104]. To complicate this, GH administration in adults with GHD or type 1 diabetes improves insulin-sensitivity without inducing lipolysis due to a predominant insulin-like action of IGF-I [105]. The fact that GH shares the same signaling network with insulin explains both the insulin-mimetic and antagonistic effect of GH [1,99].

A useful model to speculate about the effect of carbohydrates on GH levels is provided by the oral glucose tolerance test (OGTT).

Given the ability of glucose to suppress GH secretion, the OGTT is widely used to assess proper GH inhibition in healthy and pathological conditions, and is considered the gold standard method to confirm or exclude GH hypersecretion [106]. On the other hand, hypoglycemia stimulates GH release, and insulin-induced hypoglycemia can be used to assess the integrity of GH secretion [107]. Following an oral glucose administration in humans, GH shows a biphasic response: a transient suppression of plasmatic GH for 2–3 h followed by a delayed rise 3–5 h after glucose ingestion [108,109]. The exact mechanism leading to GH suppression during OGTT is unclear, and complicated by species disparities that make rats an inadequate model to study. As demonstrated in various experiment using GHRH or pyridostigmine [110,111,112], glucose increases hypothalamic somatostatin release, suppressing GH. After 3–5 h, the somatostatinergic tone decreases, allowing GHRH secretion [113]: GH accumulated in the pituitary gland is then released, explaining the delayed rebound peak.

Recently, ghrelin has been suggested as a modulator of post-glucose GH secretion [114]. As for GH, macronutrients and insulin modulate ghrelin secretion. Both intravenous and oral glucose exert a potent inhibitory stimulus [114,115,116]. Glucose-induced ghrelin decrease is coupled with a parallel decrease in GH level [114], and they share a similar circadian rhythm with a rise before the onset of meals, a postprandial decline, and a nocturnal rise [115]; more strikingly, in a multivariate analysis ghrelin was the only predictor of basal and peak GH after an OGTT [117]. When evaluating different types of carbohydrates, both oral glucose and fructose inhibit ghrelin, even if fructose is not associated with marked plasmatic glucose elevation, and the rise in circulating insulin is less pronounced than with oral glucose [78]. Even a light breakfast composed of 45% carbohydrates inhibits ghrelin similarly to the OGTT, although the rise in glucose and insulin is less pronounced [118], showing a possible modulatory role for fat and protein. The same effect of a similar mixed meal is evident in type 2 diabetic patients: when compared to healthy control exposed to oral or intravenous glucose [115], glucose-mediated inhibition of ghrelin is more rapid when carbohydrates are administered intravenously than orally. Both insulin infusion with subsequent hypoglycemia and the euglycemic clamp cause a ghrelin decrease, indicating the main regulator role of hyperinsulinemia, rather than hyperglycemia, on ghrelin [119,120]. Furthermore, evaluating the effect of isovolumetric, isocaloric beverages mainly composed of carbohydrates, lipids, or proteins, the latter resulted the most effective in reducing ghrelin, while carbohydrates resulted in the largest initial drop in the hormone. Interestingly, only after carbohydrates, there is a peak rebound of ghrelin above the pre-prandial level [121]. To complicate the matter, ghrelin infusion enhances somatotroph secretion and causes marked hyperglycemia coupled with a slight decrease in insulin level persisting for 2 h and inhibition of lipolysis, suggesting some non-GH-mediated effect on glucose [122].

#### Fibers

Dietary fiber consists of nondigestible carbohydrates that are intrinsic and intact in plants [123]. Classically, they have two main components: soluble and insoluble fibers [124]. Dietary fibers are many and include non-starch polysaccharides and other components such as cellulose, resistant starch, resistant dextrins, inulin, lignins, pectins, beta-glucans, and fructo-oligosaccharides. The recommended daily dose is 25–30 g of fiber in adults [95,96,97]. Dietary fiber intake influences several metabolic processes, including the absorption of nutrients, as well as carbohydrate, fat, and sterol metabolism [125]. Fiber fermentation at the colonic level favors a saccharolytic microbiota able to produce short chain fatty acids (SCFA) that modulate the host metabolism [125,126,127]. The role of fibers in GH regulation has never been described with precision; however, when evaluating their impact on GH secretion in stimulation tests and basal conditions, one study found a positive correlation among stimulated and endogenous GH secretion, IGF-I levels, and dietary fibers independently by confounders, although the mechanisms are unclear and still unexplored [128].

### 4.2. Amino Acids and Proteins

AAs are the basic building blocks of proteins and they serve as the nitrogenous backbones for compounds like neurotransmitters and hormones [129]. The deaminated carbon skeletons of AAs can serve as a source of energy for the human body. They are converted to intermediates that ultimately form either glycogen or fat and are accordingly classified as glucogenic or ketogenic [130]. Leucine belongs to the latter group, whereas isoleucine, lysine, phenylalanine, tyrosine, and threonine may be either ketogenic (by way of acetyl-CoA) or glycogenic. All other AAs are considered glycogenic [130]. Indeed, some AAs are used for protein biosynthesis, while others are converted to glucose through gluconeogenesis, or enter the citric acid cycle. Although proteins have pivotal functional and structural roles, promoting growth and maintenance, these molecules, especially those of muscles, are the last options under starvation conditions to provide energy and support life. The protein intake requirements are widely debated in literature and are influenced by several intrinsic and lifestyle factors; however, it is generally suggested that dietary proteins should account for ∼15% of energy when in energy balance and weight stable, with a proposed maximum approximately 25% of energy in specific conditions [95,131,132]. AAs and protein metabolism are in a close relationship with the GH/IGF-I axis due to their anabolic role.

Intravenous administration of various AAs can stimulate GH secretion [133,134]. In detail, basic AAs, such as arginine, histidine, and lysine, elicit a clear rise in GH levels when infused intravenously. Leucine and valine seemed less potent, whereas isoleucine did not appear to affect plasma GH concentrations [134]. The effect of arginine on stimulated GH secretion is dependent on the suppression of the endogenous somatostatin secretion at the hypothalamus level [135]. In addition, the combination of arginine infusion (1 g/kg up to 30 g in 30 min) and GHRH bolus is clinically used as a test to assess the responsiveness of the GH secretory system to exclude a condition of GHD [136,137].

AAs also stimulate GH secretion upon oral administration, with different potency among studies: for example, the ingestion of a physiological dose (24 g) of essential AAs increased GH concentrations 2.1-fold compared to basal condition [138] while the ingestion of glutamine or arginine led to GH increases of 2- and 4.5-fold, respectively [139,140]. The combination of arginine and lysine had the most powerful resultes, increasing GH 3- to 8-fold, depending on the study [141,142]. 

However, the GH response to AA administration may be affected by several factors such as physical training, sex, diet, time since last meal, and age [133]. In healthy young male bodybuilders, serum GH levels were not consistently altered following the ingestion of AA supplements (total 2.4 g of arginine and lysine or 1.85 g of ornithine and tyrosine over a 3- h period after an overnight fast) [143]. Similar results were reported in bodybuilders ingesting 40, 100, and 170 mg/kg of ornithine after an overnight fast: GH levels rose significantly after the highest dosage only [144], suggesting that higher amounts of AAs are needed to increase GH in well-trained adults. Notably, AAs determine a greater and more consistent increase in females than males, probably because of the synergic effect of oestrogen. In fact, a pre-treatment with stilbestrol in males led to a larger GH response to arginine infusion than without it [145]. Age is another determinant of GH response, with younger individuals showing a more robust response: arginine infusion stimulated GH secretion in all subjects aged 16–19, but in less than half (44%) of those aged 20–29, and in only 18% of those aged 30–71 [146].

Similarly to AAs, proteins affect GH secretion. For example, soy proteins stimulate GH secretion when ingested either as hydrolysed proteins or free AAs [147]. Furthermore, the acute GH response to AA ingestion may be influenced by the daily amount of dietary protein or AA consumption: diets high in proteins apparently increase basal GH levels [148]. This could be the reason why, in male bodybuilders who follow high protein diets, the use of specific AAs before exercise may not be very effective in inducing GH release [143]. Low blood glucose levels act synergically with AAs in stimulating physiologic and pharmacologic GH secretion; conditions such as a low carbohydrate diet and fasting should be kept in mind, especially latter, underlying how the timing of ingestion of AAs could modify GH response [149,150]. 

AAs have a positive effect on IGF-I secretion as well. Infants fed with formula containing tryptophan and phenylalanine showed elevated IGF-I levels compared to controls [151]. In general, it is known that high levels of proteins can promote an increase of IGF-I in muscles [152]; in particular, this association appears to be driven by animal proteins: total IGF-I levels were 13% lower in vegan women compared with meat-eaters and vegetarians, while IGFBP-1 and IGFBP-2 concentrations were 40% higher in vegan women than in meat-eaters and vegetarians, and this may be related to a reduced proportion of free IGF-I [153]. Furthermore, an increase in milk intake from 200 to 600 mL/day corresponded to a 30% increase in circulating IGF-I [154]. The positive association between milk and dairy-product intake and circulating IGF-I levels was described by several observational studies [155,156,157,158,159], although with unclear mechanisms. In animal studies, it was demonstrated that dietary proteins influence circulating IGF-I concentrations [160,161], that AA availability differentially regulates IGF-I and IGFBP-1 gene expression in primary cultures of rat hepatocytes by activation of transcriptional processes [162], and that milk-borne IGF-I could have metabolic effects on liver and other peripheral tissues [163]. Because IGF-I affects proliferation and differentiation [164], excessive milk intake has been investigated and related to several cancers, in particular of the prostate, although evidence is still limited and debated because dairy products seem to be protective [165,166].

IGF-I levels may change depending on the abundance or lack of energy substrates. In humans, CR reduces the levels of IGF-I, but this happens only if protein intake is also restricted [167]. In addition, there is a difference between protein sources (plant versus animal proteins), since diets containing low, plant-based AAs promote multiple aspects of health [168]. Similarly, soy and whey proteins increase insulin sensitivity and reduced adiposity, causing life span extension [169]. On the other hand, milk or casein proteins increase circulating IGF-I, insulin, and satiety hormones [156,170,171]. Likewise, tryptophan [171] and methionine [172] promote GH and IGF-I secretion, so diets with lower levels of these AAs, decreasing IGF-I, can promote long-term health [173]. In a condition of pathological CR, such as anorexia, salivary and serum IGF-I levels decrease, and thus IGF-I appears to be a reliable biochemical indicator of malnutrition [165,166]. In contrast, in obesity, GH secretion is diminished without a concomitant reduction of IGF-I levels [174], probably because obesity-associated hyperinsulinemia may reduce IGFBP, thus increasing IGF-I free concentrations [175].

The action of GH on AA/protein metabolism has a particularly important role under conditions of food deficiency and stress, being the only hormone to increase during fasting, while insulin and IGF-I decrease [176]. This mechanism is fundamental to preserve protein stores, in fact GH inhibits protein breakdown and stimulates protein synthesis in muscle and other tissue and inhibits AA degradation and ureagenesis in liver [1]. Probably this “defensive” mechanism is also mediated via IGF-I, considering that chronic GH exposure increased hepatic IGF-I production and suppressed IGFBP-1 [177]. Indeed, data on the acute effects of GH on protein in basal metabolic conditions needs further evaluations.

Consumption of branched chain amino acids (BCAAs) resulted in an increase of serum IGF-I levels in humans [178]. Moreover, after administration of L-leucine to trained men, IGF-I levels increased [179]. Considering pathological GH conditions, metabolomic analysis of patients affected by acromegaly suggests that the main metabolic fingerprint of GH hypersecretion is a reduction in BCAAs, related to the disease activity [180]. The mechanisms of decreased levels of BCAAs in acromegalic patients compared to general population has not been established so far, but a possible explanation could be the increasing uptake of BCAAs by the muscles, thus favoring an anabolic action. Moreover, serum GH levels are inversely correlated with valine and isoleucine in normal subjects as well as in acromegalic patients, while no association between BCAAs and IGF-I levels has been found, suggesting that GH rather than IGF-I is the main mediator of the metabolic fingerprint [180]. Thus, the low levels of BCAAs in acromegalic subjects could be related to increased gluconeogenesis and raised consumption of BCAAs. Supporting this hypothesis, a recent study, described the presence of reduced BCAAs levels in two siblings affected by growth impairment related to pregnancy-associated plasma protein A2 (PAPP-A2) gene mutation, resulting in low IGF-I bioactivity and high GH levels due to a feed-back mechanism [181]. After rhIGF-I therapy, GH levels decreased and BCAA levels increased. Interestingly, as BCAAs have been suggested as markers of insulin resistance in children, after therapy, metabolic profile improved as well. Furthermore, in GH-deficient rats, GH administration increased the expression of l-type amino-acid transporter 1 (LAT1) thus improving the transport of BCAAs into the myocyte [182].

### 4.3. Lipids and Free Fatty Acids

Lipids are polar and nonpolar compounds that include triglycerides (TGs), diglycerides, monoglycerides, fatty acids, phospholipids, and sterols, and contribute to food taste, consistency, and energetic content. Physiologically, they represent a quickly available source of energy and are fundamental as a component of different structures of cell membranes, eicosanoid, and steroid hormone precursor; they help to absorb various food components, especially fat-soluble vitamins [183]. The TG structure consists of a glycerol core and three fatty acids, which can have different lengths and grades of saturation (number of double carbon bonds in the carbon chain): saturated fatty acids (SFAs) have no double bonds, monounsaturated fatty acids (MUFAs) have one, while polyunsaturated (PUFAs) have more than one. According to the length of the carbon chain, fatty acids can be divided in short- (3–6 carbons), medium- (8–14 carbons) and long- (16 or more carbons) chain. Phospholipids are made of one glycerol molecule esterified with 1–2 fatty acids and a polar head group [183]. The digestion of TG begins with conversion to monoglyceride and two fatty acids; these are taken up by enterocytes where fats are re- assembled into chylomicrons and, to a lesser extent, very-low-density lipoproteins (VLDL), and then secreted into circulation. The liver can metabolize fats, primarily in VLDL, while adipose tissue removes lipids from chylomicrons and lipoproteins via hydrolyzation in order to absorb and store them. In healthy individuals, a small amount of fat reaches the large intestine, where is metabolized by the microbiota [183].

A close link exists between GH and lipid metabolism, since GH plays an important role in controlling intermediate metabolism, body composition, and energy expenditure. 

GH’s actions on lipid metabolism are peculiar, as, in humans, GH and FFA levels are inversely correlated. In fact, GH has a lipolytic action, while FFAs have an inhibitory effect on GH secretion, and thus a negative feedback mechanism of FFAs on somatotropic cells under physiological conditions has been hypothesized [184].

After an overnight fast, GH acts essentially by stimulating lipolysis and lipid oxidation. A single exogenous GH pulse causes a marked increase in circulating levels of FFA and ketone bodies, as a consequence of lipolysis and ketogenesis stimulation [185]. GH’s effects on lipolysis seem to be related to insulin resistance in a vicious cycle. After GH infusion, GH impairs the insulin-elicited suppression of endogenous glucose production, but this effect is not correlated to modifications of hepatic glucose-glucose-6-phosphate cycle activity, which is a marker of insulin resistance in many metabolic disorders. Thus, the increased concentration of FFAs induced by GH causes resistance to glucose utilization by incrementing insulin levels, suggesting that the glucose-fatty acid cycle may be an important mechanism contributing to the insulin resistance induced by GH [186]. On the other hand, the antilipolytic agent acipimox, which causes a reduction of FFAs, improves insulin sensitivity [187].

In addition to the effect on lipolysis, GH regulates the metabolism of triglyceride-rich VLDL and, therefore, the availability of FFAs for peripheral tissues. GH has a stimulatory action on the expression of low-density lipoprotein (LDL) receptors and the production of high-density lipoprotein (HDL)- cholesterol, resulting in the removal of circulating cholesterol. Overall, the different actions of GH on lipid metabolism result in a reduction in body-fat mass, as well as a redistribution of the fat mass with consequent protection from atherosclerotic risk [188,189]. The action of the somatotropic hormone on carbohydrate metabolism is very complex, as detailed in a subsequent dedicated section; it involves acute and late effects, direct or IGF-I-mediated actions. GH stimulates insulin release and increases glucose oxidation, resulting in a decrease in blood sugar levels, corresponding to an increased esterification of FFAs in the adipose tissue [190]. In turn, FFAs have a great inhibitory effect on GH secretion. In both animals and humans, the infusion of mixed FFAs is accompanied by the inhibition of both spontaneous and GHRH-stimulated somatotropic secretion. In particular, the latter effect is observed when GHRH is administered together with substances inhibiting the somatostatinergic tone, such as arginine, or with cholinergic agonists. On the other hand, both in animal and human models, the pharmacological reduction of FFA levels obtained by lipolysis-inhibiting drugs has been shown to increase basal and GHRH-stimulated somatotropic secretion [191]. The anti-lipolytic drug acipimox, which drastically reduces circulating FFAs [192], does not significantly increase basal GH levels compared to controls [193], unless administered in combination with GHRH at maximal doses, thus indicating that the effects of FFAs on GH are not dependent on the endogenous release of GHRH [51,52]. Whether the action of FFAs is directly mediated by pituitary secretion [52,63,194,195], or whether it is mediated by the inhibition of somatostatin release at the hypothalamic level [196], is not clear at all. Initially, animal studies supported the second hypothesis, but subsequent data did not rule out the possibility that the effect of FFAs may be mediated by somatotropic cells. In particular, FFAs could interact with cell membranes, inhibiting their depolarization and, consequently, the release of GH. Clinically, endocrinological diseases as obesity, type 2 diabetes mellitus, and Cushing’s syndrome are all characterized by chronic elevation of circulating FFAs and a substantial depression of the somatotropic function has been documented, thus supporting the hypothesis that FFAs play a prominent role in the pathogenesis of GH secretion alterations that are characteristic of these diseases [197,198,199,200].

IGF-I not only mediates the actions of GH, but also increases and amplifies the anabolic actions of GH and counteracts its harmful effects (lipolysis, gluconeogenesis, and reduction of insulin action). In vitro studies demonstrated that IGF-I has an insulin-mimetic effect on adipocytes at high hormone concentrations only, probably mediated throughout a cross-reaction with the insulin receptor, since IGF-IR is not expressed in fat cells [201]. In line with these findings, human studies showed a reduction in circulating plasma FFAs after IGF-I infusion at high rate. Considering that the primary pool of plasma FFAs is from TG accumulated in fat cells, the decrease in FFA concentration could reflect an inhibition of lipolysis [202]. 

The effects of specific FFAs on the GH/IGF-I axis are still anecdotal and often contrasting. Diets rich in alfa-linoleic acid up-regulate IGF-I, GHR, and IGFBP mRNA in the liver and IGF-I secretion. This is in line with the observation that nutrient alfa-linolenic acid deficiency is associated with poor growth [203]. In GH3 cells, the sodium salts of butyric, valerate, hexanoic, caprylic, nonanoic, and dodecanoic acids increased GH and prolactin (PRL) secretion [204]. In pre-weaning calves, the addition of sodium-butyrate to milk formula increased the secretion of GH and insulin levels [205]. In contrast, propionate and butyrate inhibited GHRH-induced GH release in goat anterior pituitary cells [206]. Similarly, in dairy cow anterior pituitary cells, acetate, propionate, and butyrate inhibited GH and PRL gene transcription via the cAMP/PKA/CREB signaling pathway [207]. In particular, β-hydroxybutyric acid decreased in vitro GHRH synthesis and secretion via the GPR109A/ERK1/2 MAPK pathway in the hypothalamus [208].

Regulation among the GH/IGF-I axis and macronutrients is summarized in Table 1.

## 5. Regulation of GH and GH Signaling from Micronutrients

### 5.1. Vitamins

Vitamins are essential micronutrients derived from the diet, that the human body needs in small quantities for the proper functioning of its metabolism. Most vitamins are groups of related molecules, classified as either water-soluble or fat-soluble. In humans, there are 13 vitamins, of which 4 are fat-soluble (A, D, E, and K). Although classified as a vitamin, vitamin D, beyond a relative intake with the diet, is also produced by the human body after sun exposure.

In the last 50 years, research focused on the crosstalk among vitamins and the GH/IGF-I axis, with the most in-depth data related to vitamin D, since it shares a role with GH in bone homeostasis.

#### 5.1.1. Vitamin D

Both vitamin D and the GH/IGF-I axis are fundamental to skeletal growth and bone maintenance. Vitamin D seems to influence the GH axis at multiple levels. At the pituitary level, GH-secreting cells express the vitamin D receptor (VDR). It is likely that 1,25(OH)2D3, the active form of vitamin D, binds to the human pituitary VDR stimulating GH secretion, modulating the expression of several genes and factors, including pituitary transcription factor-1 (Pit-1), and contributing to the multifactorial etiology of GHD [209,210,211]. In the liver, stellate, Kupffer, and endothelial cells express VDR, which could regulate IGF-I synthesis and secretion. 1,25(OH)2D3 likely promotes liver production of IGF-I and IGFBP-3 by directly inducing their transcription and/or by enhancing GH stimulation [212]. However, although IGF-I, IGF-IR, and IGFBPs are vitamin D target genes [213], data are still controversial [214]. Surely, in epiphyseal chondrocytes, 1,25(OH)2D3 potentiates IGF-I synthesis and stimulates cell differentiation through mediation by local IGF-I synthesis [215]. Furthermore, mice knocked out for the VDR have lower IGF-I levels than control littermates [216], and in mice knocked out for steroid receptor co-activator 3 (SRC-3), one of the co-activators of VDR, liver expression and circulating levels of IGFBP-3, and consequently IGF-I, are decreased [217]. Vitamin D also could increase IGF-I levels by increasing calcium absorption in the gut, according to results on diets rich in calcium in VDR-knocked out mice [216].

Vitamin D levels, considered variably as 25(OH)D3 or 1,25(OH)2D3, are often lower in patients with GHD, as well as with acromegaly, than in healthy controls, while the impact of GH treatment on vitamin D levels remains not definitive [214]. What is clear is that circulating or locally-produced IGF-I results in 1,25(OH)2D3 and 24,25-(OH)2D3 production in the renal tubules [212,218] and placenta [219], suggesting that 1α-hydroxylation of 25(OH)D3 is not exclusively under the control of calcium, phosphate, PTH, and fibroblast growth factor 23 (FGF23). 

Further research is needed to decipher how vitamin D metabolites and the GH/IGF-I axis are intertwined in different tissues that are the target of the three hormones.

#### 5.1.2. Vitamin A

Vitamin A has multiple functions, including growth and development. Vitamin A is often used as a collective term for several molecules called retinoids. In the retinoic acid form, it plays an important role in gene transcription, and one of its receptors, RXR, can heterodimerize with VDR.

Interestingly, vitamin A modulates the GH gene through its interaction with RXR-α, the predominant receptor form in the pituitary [220]. Accordingly, mutations within the retinoic acid response element of Pit-1 cause hypopituitarism and GHD, as well as deletions within the RXR- α gene [221]. In vitro, vitamin A also possesses non-transcriptional effects, increasing GH secretion within minutes [222]. In the liver of vitamin A-deficient rats, GH-regulated CYP2C11, CYP4A2, and IGF-I are down-regulated, and GH-responsiveness of the JAK-STAT system is reduced [223]. Epidemiologic studies have shown that children with short stature have frequently lower vitamin A intake than those with normal stature [224], and a close correlation between plasma vitamin A and nocturnal GH secretion exists in children with neurosecretory dysfunction [225]. However, the effects of vitamin A deficiency and GHD or GH administration on bone growth and architecture is far from being clarified.

#### 5.1.3. Vitamin E

The most important function of vitamin E molecules is their antioxidant activity to maintain the structural integrity of our cells. Only a few studies investigated its role on the GH/IGF-I axis, showing increased oxidative stress with low vitamin E levels in GHD children [226]. No mechanistic studies have been done to our knowledge.

#### 5.1.4. Vitamins of the B Complex

Vitamins of the B complex are essential water-soluble vitamins that regulate a multitude of cellular processes in vertebrates, including growth, development, and oxidative processes. Several studies have investigated the role of vitamin B6, 8, and 12 concerning GH secretion and growth.

Pyridoxine (vitamin B6) induced PRL secretion in healthy humans, whereas pyridoxal phosphate, its bioactive form, decreased GH secretion in acromegaly and infants, but not in other conditions. In vitro studies demonstrated that this effect is mediated by inhibition of cell proliferation, hormone secretion through action on cell-cycle arrest, and apoptosis [227].

Biotin (vitamin B8) dietary restriction is associated with body growth and size. This phenotype is indeed associated with a decreased availability of IGF-I without changes in GH circulating levels in mice [228].

Several carriers of vitamin B12, as intrinsic factors, haptocorrin, and transcobalamin II were up-regulated in dwarf rats treated with GH, suggesting that it increases the availability of vitamin B12 to be used for several functions, mainly thymidine synthesis [229]. Interestingly, an elegant study increased the knowledge on this topic, suggesting a novel gut/bone/liver axis. The authors demonstrated that vitamin B12 deficiency results in severe postweaning growth retardation and osteoporosis in a mice model. This condition is associated with a decreased production in the liver of taurine, a (semi)essential amino acid critical for growth and metabolism, resulting in an abrogation of the GH/IGF-I axis with a condition of GH resistance. Indeed, GH regulates taurine synthesis in a STAT-5 and vitamin-B12-dependent manner. Furthermore, taurine is an up-stream regulator of IGF-I synthesis in the liver and action on osteoblasts [230]. 

### 5.2. Minerals

In the context of human nutrition, minerals are chemical elements required as essential nutrients derived from the diet (foods and water) to perform functions necessary for life, supporting the biochemical reactions of metabolism. The more abundant minerals in the human body are calcium, phosphorus, potassium, sodium, and magnesium; the others, equally important, are the so-called “trace elements” sulfur, iron, chlorine, cobalt, copper, zinc, manganese, molybdenum, iodine, and selenium. Being involved in human growth, bone accretion, and body maintenance, the GH/IGF-I axis is strictly involved with all the minerals, with some closer relationships with water balance and zinc.

#### 5.2.1. Sodium, Potassium, and Water

Osmotic stimuli, such as dehydration, after the hormonal fast responses, is followed by the increased secretion of long-term acting hormones, such as GH, to bring about increased protein synthesis, cell proliferation, differentiation, and tissue reorganization, allowing increased transport capacity in the acclimation phase of salt demands. In mammals, GH affects salt and water retention, acting on tubular renal function, as clearly demonstrated by conditions of GHD or GH excess [231]. IGF-I, through the modulation of renin release, inhibition of atrial natriuretic peptide (ANP), and activation of distal tubular sodium (Na) channels, participates in glomerular and tubular Na retention up to 50% [232,233]. Both GH and IGF-I seem to synergistically stimulate the transepithelial Na transport on cortical collecting duct cells, with a complex final modulation of the epithelial Na channel (ENaC) [231]. The action of IGF-I on the Na-phosphate cotransporter seems to not be fundamental to increased Na retention [232]. Accordingly, transient retention of water and NaCl due to an increase in extracellular volume is observed in GH- or IGF-I deficient subjects who start a treatment with recombinant GH or IGF-I [232,234,235]. Some authors suggest that this transient Na overload might, in theory, be prevented by the coadministration of the ENaC blocker amiloride [231]. Furthermore, acromegalic patients had a larger amount of total body water and extracellular volume and an increased renal ENaC activity [231,235,236]. Although the mechanisms for GH and IGF-I seem similar and redundant in renal electrolyte and water handling, this is not completely true, because GH stimulates renal water reabsorption, while IGF-I does not affect this process.

Urinary potassium excretion seems transiently decreased in patients within a day after initiation of GH-administration, although these findings remain controversial. Diversely, it is well demonstrated that animal models of potassium depletion are characterized by rapid kidney growth, and increased intrarenal IGF-I levels, with concomitant low circulating and hepatic IGF-I levels that impaired animal growth [232,237,238].

#### 5.2.2. Calcium

The relationship between calcium (Ca^2+^) and the GH/IGF-I axis is very close, as GH signaling requires Ca^2+^. Increasing evidence suggests that L-type Ca^2+^ channels have a major role in Ca^2+^ influx in pituitary cells, and both basal and stimulated GH secretion is dependent on this Ca^2+^ influx [239,240]. Therefore, Ca^2+^ channel blockers may be useful in the treatment of acromegaly [240]. However, the relationship between GH and IGF-I and the mineral is probably U-shaped rather than linear; in fact, elevated serum Ca^2+^ and PTH concentration are associated with low IGF-I levels and a blunted response of GH to stimuli [112,241]. Although the mechanisms leading to reduced GH secretion remain to be clarified, extracellular Ca^2+^-sensing receptor are expressed in the human pituitary and involved in the amplification of the GH response to GHRH [242]. Interestingly, long-term total parenteral nutrition was associated with hypercalciuria, hypercalcemia, growth retardation, and low IGF-I levels in a six-year-old child with an improvement in growth velocity by reducing Ca^2+^ content [243].

By considering the somatotroph axis, GH and IGF-I play a crucial role in adapting Ca^2+^ homeostasis, mainly in periods of bone growth, characterized by increased demand for it. GH, through IGF-I, acts on Ca^2+^ gut absorption mediated by the epithelial Ca^2+^ channel TRPV6. This action is independent of the GH/IGF-I regulation of 1,25(OH)2D3 secretion in the kidney [231,244]. Furthermore, at the kidney level, IGF-I stimulates 1,25(OH)2D3 production to increase Ca^2+^ reabsorption by increasing the TRPV5 channel in the distal tubule [231,245]. Indeed, in the condition of GH hypersecretion and GHD, Ca^2+^ handling is subject to modifications. The effect is particularly relevant in acromegalic patients, in whom IGF-I-mediated 1,25(OH)2D3 production results in absorptive hypercalciuria and increased fasting plasma Ca^2+^ [231,246].

#### 5.2.3. Phosphorus

Normal phosphate homeostasis is also essential for normal linear growth and correct bone mineralization. Restriction of dietary phosphorus needs a functional GH/IGF-I axis to lead to correct production of 1,25(OH)2D3 as a feedback mechanism [247,248]. In GHD, phosphate tubular reabsorption is reduced, resulting in a relative phosphate-deficient state. Treatment with rhGH raises the renal tubular phosphate absorption in both normal and GHD subjects [249,250]. Indeed, GH induces a positive phosphate balance due to enhanced renal phosphate absorption acting on the Na-phosphate cotransport. IGF-I also reduces renal phosphate excretion up to 49% when administered to normal subjects, also in chronic conditions, through a Na-phosphate cotransporter [251,252,253]. Whether the action of GH is completely dependent on IGF-I is still a matter of debate; however, these GH and IGF-I actions seem independent of PTH regulation [232,254,255]. Recent data suggest that the increase in serum phosphate under GH treatment or acromegaly is unexpectedly associated with upregulation of the phosphaturic FGF23/Klotho axis, suggesting counter-regulatory mechanisms or an FGF23 resistance induced by GH-stimulated Klotho secretion in the kidney [250,254,255].

#### 5.2.4. Magnesium

Magnesium (Mg), as free Mg^2+^, modulates the activity of hormone-sensitive adenylate cyclase systems in many tissues, including somatotroph cells. Free Mg^2+^ in cytoplasm seems critical for the transformation of the cyclase to its high activity state and preventing unnecessary synthesis of cAMP [256]. Interestingly, nutritional deficiency of Mg in young rats was associated with growth retardation and lower circulating IGF-I levels, up to 60–76%, without changes in stimulated GH secretion, independently by food intake. Diet repletion of Mg restores IGF-I levels, weight, and length growth [257]. Because GH secretion is not impaired, a reduction in IGF-I caused by GHR or post-receptor defects has been hypothesized. Blood pH seems to have a role in the balance between Mg and GH action. GH administration in humans blunts renal Mg wasting induced by an experimental metabolic acidosis, with attenuated hypomagnesemia and hypermagnesuria, whereas Mg excretion is unchanged after prolonged GH administration in non-acidotic subjects [258,259]. Because gut Mg absorption is not modified by GH in humans, the observed effect in acidosis is supposed to be the results of a hyperresponsive bone accretion after a previous period of bone loss [260].

#### 5.2.5. Zinc

Zinc (Zn) is required for the function of over 300 enzymes and 1000 transcription factors, and is a messenger that activates several signaling pathways. Indeed, the biological roles of Zn are ubiquitous in the human body and essential for human growth. Its homeostasis is mainly controlled by the gut. Zn deficiency has been implicated in growth inhibition, gastrointestinal disorders, and several chronic diseases [260,261].

Zn nutritional status has been demonstrated to deeply influence the GH/IGF-I axis. Zn regulates the signaling ability of GH by interacting with GH itself and favoring its dimeric form, which is more stable than the monomeric one [262,263]. Furthermore, it seems to prolong the binding of GH to the GHR, lengthening the signal transduction and to favor GH aggregation in secretory granules in the somatotroph cells [264,265]. Furthermore, Zn deficiency could influence the Zn- proteins target of the GH/IGF-I actions in several tissues including pituitary, liver, and adipose [221,266,267].

Dietary Zn and proteins are two of the main determinants of IGF-I synthesis [260,261]. In rats, nutritional Zn deficiency results in decreased body weight; serum IGF-I, GH, and GHBP concentrations, and hepatic GHR and IGF-I expression [268,269]. Accordingly, Zn supplementation increased GH, IGF-I, and IGFBP-3 levels in animal models [260,269], as well as in GHD and healthy children [270,271,272]. The supplementation was also associated with increased consumption of dietary proteins and fats [272]. Because Zn potentiates the anabolic effect of IGF-I in osteoblastic cells with a complex network also including other essential minerals critical for the calcification of the bone matrix, Zn supplementation in GHD children also improves bone mineral content and area [261,271,273]. This is in line with evidence that GH treatment fails to act on bone growth in Zn-deficient animals, likely due to poor Zn availability in the bone cells [267].

#### 5.2.6. Iron 

Iron deficiency has been implicated as a cause of stunting, developmental delay, and alterations of immunity functions that is beyond anemia because iron is an essential component of hemoglobin. Interestingly, iron and Zn share the same digestive transport mechanisms, competing during gut absorption. Some authors suggest that iron status, more than Zn status, is correlated with growth [274]. While data on iron regulation on GH/IGF-I axis are few, the GH/IGF-I system does act on several mechanisms of the iron pathway. As an example, hemopexin, an essential heme scavenger produced in the liver to re-utilize iron, is a GH-inducible gene [275]. Interestingly, IGFBP-3, which increases the half-life of IGF-I and exerts IGF-independent modulation of growth, is also able to bind to transferrin, the primary iron-carrying protein in serum. This binding could be another mechanism to influence growth and GH/IGF-I homeostasis [276].

In vitro and in vivo evidence suggests an independent erythropoietic effect of IGF-I through IGF-IR in both precursors and mature erythrocytes, stimulating proliferation and differentiation, and modulating protoporphyrin synthesis [277,278]. Indeed, low IGF-I levels are one of the causal factors of anemia in both children and adults [279], and GH treatment decreased ferritin concentration and increased serum transferrin concentration indicating erythropoiesis [280].

#### 5.2.7. Iodine

Iodine is required for the synthesis of the growth-regulating thyroid hormones. Iodine deficiency in utero impairs fetal growth, and iodine supplementation during pregnancy increases birth weight by 100–200 g [281]. Similar effects on growth are reported in school-age iodine-deficient children [282]. This effect is partly due to direct effects of thyroid hormones on epiphyseal growth, bone maturation, and stature, but also on the GH/IGF-I axis [215]. Thyroid hormones are important for normal GH expression in vitro and in vivo, and in feedback mechanisms on the GHR [282,283]. Furthermore, blood levels of IGF-I and IGFBP-3 are dependent on thyroid function [282,284] through GH-mediated effects and direct stimulation as well [285]. In moderately- severe iodine-deficient children, IGF-I increased by 50–100% after 6–10 months of iodine supplementation [282]. The role of iodine supplementation in tuning the GH/IGF-I axis in healthy or GHD/acromegalic adults is less explored.

#### 5.2.8. Selenium

Selenium is critical for some antioxidant enzymes, including glutathione peroxidase, protecting from the adverse effects of free radicals. Selenium has been studied since 1860 for the so-called alkali disease. Selenium intoxication in animals is characterized by growth retardation because it can accumulate in the anterior pituitary and liver, altering both somatotroph and liver functions. In this condition, the GH response to GHRH is blunted and IGF-I levels do not substantially increase after withdrawal of selenium and concomitant rhGH treatment [286,287]. Accordingly, selenium administration reduces the expression of the IGF-I receptor and its signaling pathway in several tissues [288,289], supporting the hypothesis of a protective effect of selenium against cancer. However, the role of selenium on the somatotroph axis seems to be U-shaped. Selenium deficiency has been shown to be associated with growth retardation and markedly low pituitary GH and circulating IGF-I concentrations. This condition could result from a decreased activity of the type II deiodinase selenoenzyme in the pituitary, an enzyme critical for adequate GH synthesis. Moreover, because primary dietary sources of selenium are proteins, an impaired dietary protein absorption could be another causative factor. An alternative hypothesis derives from inflammation secondary to selenium deficiency with higher IL-6 levels that in turn could negatively modulate IGF-I secretion [290,291]. This observation finds a relation with the direct association between circulating IGF-I and selenium concentration in humans [292,293,294]. Although this is intriguing data in animals and cross-sectional studies, randomized control trials on the effects of selenium administration on GH-IGF-I levels are few, or suffer from methodological weakness [295].

#### 5.2.9. Manganese

Manganese (Mn^2+^) is present as a coenzyme in several biological processes, including growth. Several studies suggest that Mn^2+^ may be a master regulator of insulin/IGF-I homeostasis and signaling because it is an essential player in carbohydrate metabolism, and many kinases are Mn^2+^- dependent. Nutritional deficiency of Mn^2+^ in experimental animals results in decreased body weight and growth, and bone abnormalities without alteration in feeding behaviors [296,297]. These animals show an alteration of IGF-I metabolism, with lower IGF-I and insulin levels (up to 66% and 60%, respectively), and increased GH and IGFBP-3 as feedback mechanisms, than controls [298]. Mn^2+^ bioavailability contributes to impaired IGF signaling and glucose uptake in animal models of Huntington’s disease. In fact, Mn^2+^ is a cofactor that potentiates the effect of IGF-I on IGF-I and insulin receptor-dependent AKT phosphorylation [298]. Furthermore, Mn^2+^ increases IGF-I gene expression and IGF-IR protein expression in the medial basal hypothalamus during prepubertal female development in rats [299].

#### 5.2.10. Copper

Copper is an essential trace element for growth and bone elongation and maintenance. It is well known that high dietary copper intake promotes growth performance in pigs [300]. In rats, nutritional deficiency of copper results in low serum IGF-I levels but high IGF-I in bones, suggesting that regulatory factors that affect IGF-I hepatic synthesis are different from other tissues [301]. Furthermore, copper supplementation increases IGF-I and IGFBP-3 concentrations in culture media of chondrocytes, promoting their proliferation [289]. The effects of copper on growth could also be mediated by enhancing GHRH, GH, and ghrelin secretion, and decreasing somatostatin expression in the hypothalamus [300,302]. 

#### 5.2.11. Chromium

The precise mechanisms of chromium activities have not been fully defined, although it seems to have a role in insulin action. Chromium has been observed to have negligible or quite significant effects on GH secretion in pigs in relation to its chemical or nanotech-modified form [303].Regulation among the GH/IGF-I axis and micronutrients is summarized in Table 2.

## 6. Regulation of GH and GH Signaling in Diets and Dietary Habits

The previous paragraphs suggest how macro- and micronutrients exert a complex orchestration on the GH/IGF-I axis, suggesting that specific food regimens could have different feedback mechanisms. However, small numbers of studies were designed to determine whether variations in diet composition could modify the secretion of GH/IGF-I axis, most of all on relatively unpowered populations. 

### 6.1. Isocaloric and Hypercaloric Diet Regimens

The effects of different isocaloric and hypercaloric diet regimens on the somatotropic axis are not univocal and results should be interpreted considering the small sample of subjects included in the studies. The comparison of the effect of three isocaloric diets (high in carbohydrates or proteins or lipids: (a) 2300 kcal, 80% carbohydrate; (b) 2300 kcal, 75% high-fat; (c) 2300 kcal, 70% high-protein; and two hypercaloric diets: (d) 3600 kcal, 80% high-carbohydrate; and (e) 3600 kcal 40% carbohydrate, 40% fat, 20% protein, followed for 10–12 days by 15 men and women demonstrated that both isocaloric and hypercaloric diets high in carbohydrates reduced GH concentrations, but only in men, while high fat and protein diets had no effect on the somatotropic axis [304]. In line with the effect of carbohydrates, an isocaloric low carbohydrate, high protein diet (60% of total energy from carbohydrate, 30% fat, and 10% protein) for 7 days on 8 healthy subjects markedly reduced insulin, GH, free IGF-I, and IGFBP3 concentration, while increasing skeletal muscle IGF-I mRNA expression [305]. Diversely from the previous studies, the effects of four diets high in carbohydrates and fats at a maintenance level: (a) 62% high carbohydrate, 31% fat, 7% protein; (b) 46.5% high fat, 46.5% carbohydrate, 7% protein or 75% of maintenance energy requirements; (c) 60% high carbohydrate, 30% fat, 10% protein; and (d) 45% high fat, 45% carbohydrate, 10% protein followed for 14 days by 6 healthy males showed no significant changes in GH concentration in either fasting or fed states [306]. Furthermore, the effect of macro- and micro-nutrients intake on stimulated GH secretion among subjects with a wide range of body mass index (BMI) showed no correlation between carbohydrate intake and GH when assessed by standard stimulation test or by overnight frequent blood sampling, while a significant association between vitamin C and stimulated GH peak was found when corrected for all confounders [128]. Furthermore, no correlation was found between macronutrient intake and GH and IGF-I over a 1-year period in free-living elderly. Hormone concentrations were consistent throughout the year despite a significant difference in carbohydrate intake during the time-period examined [307]. Indeed, some authors suggest correlations between GH and carbohydrate percentages in diet regimens, although BMI and age-related variations in several studies did not confirm these findings, underlining the need for further powered studies. 

### 6.2. Caloric Restriction Regimens

In recent years, results from studies aiming to find a link between metabolism and aging demonstrated that CR, defined as a reduction in calorie intake below usual ad libitum intake without malnutrition, caused metabolic and molecular modifications in components of the nutrient-sensing and stress-responsive pathways, such as GH/IGF-I signaling, mTOR pathway, adenosine 5′-monophosphate–activated protein kinase (AMPK), forkhead box protein O (FOXO), sirtuins, and nuclear factor erythroid 2-related factor 2 (NRF2) [308,309,310,311].

The attractive interest in CR came from observations that these long-term regimens are associated with longevity in animals, and realistically in humans, by improving markers of health such as the decrease in body weight, metabolic rate, and oxidative damage [312], decreased activity of the insulin-Akt-FOXO signaling pathway [313,314], thus leading to a low incidence of non-communicable diseases.

Among the different variables that can affect the mechanisms proposed to explain metabolic and oncologic protection of CR, including the age at which CR begins, the severity of CR, and genetic background, IGF-I signaling and mutations affecting the somatotropic axis play a critical role. In fact, a reduced GH/IGF-I signaling is linked to prolonged survival and decreased incidence of cancer and type 2 diabetes in humans. A reduced IGF-I signaling suppresses the ageing process through the activation of FOXO and mTORC1 inhibition, which occur as a result of Akt inactivation [315,316]. Coherently, in animal models suppression of the insulin/IGF-I/mTOR pathways is associated with an increase in life span [317,318]. Correspondingly, GH deficient or GH resistant mice, having reduced IGF-I levels, are characterized by longer life expectancy [319]. Reduction in food intake generally reduces GH release, and the impact of reduced GH levels on circulating IGF-I under conditions of diminished energy intake is amplified by reduced responsiveness of the liver to GH signals.

The proposed downstream mechanisms leading to reduced IGF-I and improved longevity by CR are multiple. Reduction in IGF-I levels following CR is accompanied by a consensual increase in corticosterone levels as a compensatory effect for the absent metabolic role of GH, and together these effects could justify the anti-tumoral effect of CR on animals [318,320]. Moreover, a 60% CR in animals causes an increase in IGFBP-1 levels that, in turn, results in reduced IGF-I active molecules [321]. According to a randomized clinical trial in non-obese adults, the main effect of a balanced 25% CR (55% carbohydrates, 15% proteins, 30% fats) on the GH-IGF-I pathway is not a reduction of IGF-I concentration, but an increase in IGFBP-1 levels [322]. 

The modified expression of genes codifying for sirtuins, implicated in the shift from growth and reproduction to maintenance and repair, could represent a molecular mechanism for the CR-elicited suppression of the somatotropic axis, thus providing an evolutionarily explanation under nutritionally challenging or adverse conditions [323]. 

The impact of CR on the somatotropic axis is complex and time-dependent. In rodents, short term moderate 60% CR decreases GH levels, whereas long term CR preserves the pulsatile GH release [324], probably by delaying age-related modifications in the hypothalamic setting of GH secretion [325].

Studies on hypopituitary Ames dwarf mice demonstrated that GH and CR both influence longevity with different but overlapping mechanisms: a 30% CR starting at 2 months of age resulted in increased longevity, but this regimen had little effect on GH-resistant GHR-KO male mice and little effect on female mice [326]. In support of this, a recent study on domestic cattle evaluated the relationship between GH and nutrient availability: when ad libitum food intake is available, there is a selection of a genetic variant, the so called “demanding allele”, which is particularly fit to promote growth under this condition but is detrimental in the condition of CR, while the “thrifty allele” favors growth under CR, underlining how dietary regimens tend to optimize genotypic fitness to nutritional conditions [327]. 

Despite this gathering of evidence on animals, the role of IGF-I and IGF-I modifications during CR in humans is less clear. 

GH/IGF-I levels are inversely associated with cardiovascular diseases, while lower IGF-I levels are linked to reduced incidence of oncologic disease [328]. IGF-IR polymorphisms causing high IGF-I levels and reduced activity of IGF-IR are associated with longevity in centenarians [329]. In line with this evidence, studies in GHD patients demonstrated less incidence of age-related disorders as cancer and type 2 diabetes mellitus with the increasing of age [330].

Unlike rats, in humans, 1-year 20% CR has little or no effect on circulating GH and IGF-I levels unless protein intake is substantially reduced. The same results were obtained for longer (6 years) CR, while long-term protein restriction (0.76 g kg/day, ~10% of intake) reduced IGF-I levels and IGF-I:IGFBP-3 concentration [331]. 

Among CR dietary patterns, the Okinawa diet is a plant-derived low-protein diet, mostly including vegetables, fruits, and grains. Inhabitants of the Japanese island of Okinawa are the longest living people, and include five-fold more centenarians than other developed nations [332]. Factors contributing to longevity include genes, physical activity and, particularly, food quality and mild CR. Diet energy derives 9% from protein and 85% from carbohydrates [333]. The protein to carbohydrate ratio is very low (1:10), and similar to those used in recent animal longevity models [334]. In support of this hypothesis of a role of low protein intake, animal and human studies demonstrated that the reduction of protein intake, replaced by carbohydrates, could influence aging. In fact, in mouse models, BCAAs are activators of mTOR; thus, low protein intake results in reduced levels of BCAAs and ensuing mTOR inactivation [335]. Nevertheless, interactions among BCAAs, glucose metabolism, and aging signaling are complex and not completely understood yet.

The first confounder is that different CR dietary patterns exist. While caloric energy restriction (CER) has been investigated for many years, a more recent interest is in intermittent energy restriction (IER), a regimen that consents to restrict energy intake every other day with a fasting interval of 20–36 h [336]. However, the IER protocols used in most human [337,338,339] and animal [340,341,342] studies allow a small amount of food intake during fasting (modified fasting), so that energy intake is partially (≥70%) but not completely restricted, and fasting could be total or partial. The most studied approaches are the “alternate day modified fasting” (alternate days of 75% ER or two consecutive days) and the “5:2 diet” (two consecutive days of 70% ER per week) although other regimens exist [343,344]. Intakes on “non-fasting” (or “feed”) days among these studies have included ad libitum [343,345], hypoenergetic (~15–30% of energy requirements) [346,347], isoenergetic [338,348], or hyperenergetic (~125–175% of energy requirements) [349]. More tailored studies on different CR regimens in relation to protocols and macronutrient composition are needed to decipher how molecular pathways, including the GH/IGF-I axis, are affected.

Considering the biochemical model of fasting, the ketogenic diet (KD) is a high-fat, adequate protein, and low-carbohydrate dietary pattern, characterized by reducing carbohydrates to less of 10% of energy [350]. This restriction leads to a systemic shift from glucose to fatty acid metabolism, thus producing ketone bodies, such as acetoacetate and β-hydroxybutyrate, used as substrates for energy. Energy results from fat introduced by diet and by utilization of body fat. The composition of the KD is calculated by the fats/ (proteins + carbohydrates) ratio, which, in traditional versions, ranges from 3:1 to 4:1. Thus, the 4:1 KD is composed of 90% fat, 7% protein, and 3% carbohydrates. As ketone bodies replace the use of glucose by the brain, the KD has been considered for treatment of drug resistant epilepsy since 1921. In recent years, the very-low-calorie-ketogenic diet (VLCKD) has been successfully proposed for the management of obesity and metabolic disorders [351]. Interestingly, considering the assumption that ketosis decreases portal insulin levels, down-regulating hepatic GHR and thus reducing IGF-I synthesis, a 2-weeks eucaloric very-low-carbohydrate ketogenic diet (35 g of carbohydrates, ~155 g of fat, ~115 g of protein/day) has been used in patients with uncontrolled acromegaly as adjuvant treatment to first-generation somatostatin receptor ligands. KD reduced IGF-I concentrations from 1.10 to 0.83 times the upper limit of the normal range without increases in GH concentration [352]. The mechanism of this effect is not completely understood yet, and we speculate that could involve a complex crosstalk among ketone bodies, lipolysis, and release of FFA.

Among the potential long-term complications of the KD, there has been growing concern about the KD’s impact on growth in children. Since KD has been primarily used as a treatment option for children with refractory epilepsy [353], all of the studies investigating the impact of KD on children’s growth have been conducted in patients with drug-resistant epilepsy. Linear growth may be impaired on KD, especially during long term (>6 months) KD, although data are still conflicting. While several studies reported a significant decline in height or height and weight with a prolonged (1–2 years) KD [354,355,356,357,358,359,360] some authors claim that only a minority of these patients (≤30% of the population study) treated with a KD show growth deceleration/deficiency on long-term follow-up [361,362,363,364,365]. Potential mechanisms leading to poor growth in children on a KD may include chronic metabolic acidosis/ketosis in those on diets with high, i.e., 4:1, ketogenic ratio [354,355], inadequate calorie prescriptions [356,357], imbalance in the protein-to-energy ratio (<1.5 g protein/100 kcal) [366], effects of the underlying diseases and anti-epileptic drugs [355,358], and KD-induced alteration in the GH-ghrelin/IGF-I axis. In line with the latter hypothesis both IGF-I and ghrelin levels are rapidly decreased and then stabilized at low levels in children receiving a KD, and these changes were associated with poor growth indexes [355,360]. It has also been speculated that IGF-I reduction in the KD may be secondary to reduced IGF-I bioavailability due to a KD-induced “starvation-like” state that could alter IGFBP levels [355,358]. Importantly, studies characterized by a low ketogenic ratio, in which children were not calorie-restricted and received an adequate protein amount, as well as vitamin and mineral supplementation, did not report a significant negative impact of KD on growth or IGF-I levels [364,365,367]. However, further research is required to clarify the mechanisms underlying the relationships between the KD, GH/IGF-I axis alterations and growth delay.

### 6.3. Mediterranean Diet

Few data exist about the influence of Mediterranean dietary pattern on the somatotropic axis. The traditional Mediterranean diet is characterized by the consumption of a high intake of extra-virgin olive oil, fruits, cereals, nuts, legumes, and vegetables; a moderate to low intake of fish and seafood, eggs, white meat, and dairy products; and a low intake of red and processed meats, and sweets [368].

A recent cross-sectional observational study of 200 adult women with severe obesity demonstrated that the there is a positive correlation between the degree of adherence to the Mediterranean diet and protein intake and the GH peak in response to GHRH + arginine infusion. A low adherence to this diet (a score ≤5.0 with the PREDIMED score), hence a worst body composition and cardiometabolic profile, is associated with a blunted GH peak response and/or IGF-I deficiency and an alteration of the somatotropic axis is present in people that consumed high quantities of sugars and total fats, and a low amount of proteins. Since there is a well-known positive feedback on the somatotropic axis, a cluster of food and bioactive compounds typical of the Mediterranean diet could have a further synergist role to that of proteins on GH release [369].

Considering the opposite clinical condition related to GH dysregulation, no specific studies on the effect of the Mediterranean diet on IGF-I in acromegalic patients have been conducted. Nevertheless, the main metabolic consequence of acromegaly is insulin resistance, which could become overt diabetes mellitus, even if with reduced total body fat, in particular in the liver. On the contrary, fat is accumulated in less specific organs as muscle, and elevated levels of circulating lipid intermediates are detectable [370]. This leads to a vicious cycle as the stimulation of lipolysis worsens insulin sensitivity and impairs beta-cell function. Lipid and glucose metabolism is restored by curative surgery [371]. Thus, a Mediterranean diet in these patients could be effective in reducing cardiovascular risk [372] and in influencing GH status [369] but further studies are needed.

Beneficial effects on mortality rates of coronary heart disease and thrombotic stroke has been demonstrated in middle-aged men and women regularly drinking a moderate amount of red wine [373]. In fact, red wine had high flavonoid content with anti-oxidant effects [374]. Some authors [375] studied the effects of red wine in 26 healthy centenarians (nine men and 17 women, age range of 100–105 years). The subjects were subdivided in three groups according to red wine consumption and dietary habits: (a) those who had maintained the style of their dietary habits as compared to the previous years; (b) those who consumed a diet that was deficient compared to that of the previous years but remained moderate drinkers of red wine; and (c) those who consumed a diet that was deficient compared to that of the previous years and were abstainers in wine consumption. The results showed a reduction of IGF-I from group “a” to group “c”, as well as a reduction of total anti-oxidant capacity. The authors speculated that the reduced antioxidant capacity in centenarian moderate drinkers and also in abstainers could be related to metabolic deficiency, in comparison to younger people [376].

**Figure 1:**
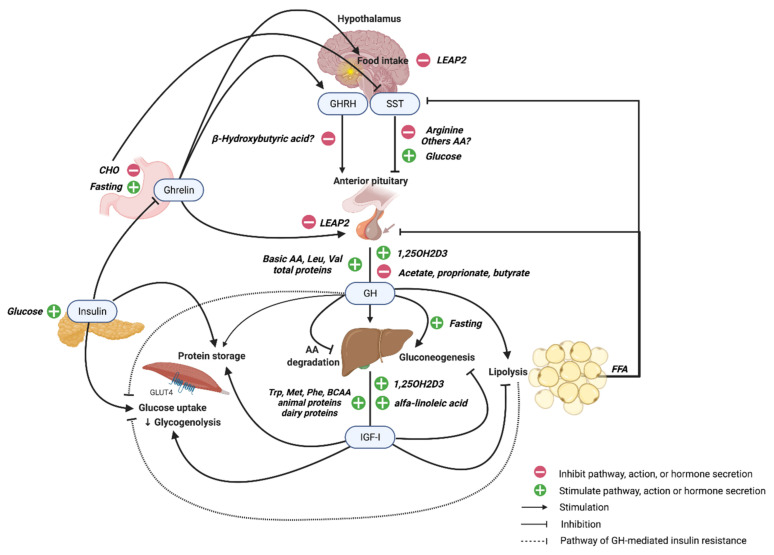
Regulation among the GH/IGF-I/ghrelin axis and macronutrients, fasting, and vitamin D. Created with BioRender.com. SST = somatostatin; CHO = carbohydrates; AA = amino acids; Leu = leucine; Val = valine; Trp = tryptophan; Met = methionine; Phe phenylalanine; BCAA = branched chain; FFA = free fatty acids; 1,25(OH)2D3 = calcitriol.

Regulation among the GH/IGF-I/ghrelin axis and macronutrients, fasting, and vitamin D is presented in Figure 1.

## 7. Healthy Eating Patterns for Patients with GH-Related Clinical Conditions: Are We Ready to Recommend a Personalized Diet?

As shown in our review, the relationship among macronutrients, micronutrients, and GH/IGF-I secretion is very tight. Nutrients and minerals contribute to the positive and negative feedback mechanisms of GH and IGF-I secretion, suggesting that in GHD and acromegaly a tailored diet is a further strategy in association with drug treatments, that contributes to stimulating or blunting GH and IGF-I secretion. A balanced intake of nutrients should be respected in daily/weekly meals to reduce the risk of each deficiency or excess. Classically, a healthy balanced diet for the general population is considered to be composed by carbohydrates for at least 45–60% of total dietary energy intake, with an amount of sugars less than 10–15% of total energy intake, proteins for 15%, and fats for 30–35% (10% saturated, 10–15% monounsaturated, and 10% polyunsaturated), avoiding trans-fats. The recommended daily dose of fibers is 25–30 g in adults [95,96,97]. However, some authors recently suggested that personalized nutrition or precision nutrition in some conditions and diseases could have an impact on the phenotype, combining dietary recommendations with individual’s genetic makeup, metabolic and microbiome characteristics, and environment [377].

Specific studies on precision nutrition in GHD and acromegaly are still in a neonatal era, and the following suggestions derived from findings summarized above should be supported by further powered clinical trials.

### 7.1. GHD

GH and IGF-I secretion are potentiated by many nutrients. Patients with GHD also suffer from visceral adiposity and metabolic diseases that need attention [137,221]. A Mediterranean diet seems to be a good choice for these patients, as it is being able to favor GH secretion [369]. In a high adherent Mediterranean diert, several nutrients should be promoted, such as proteins, mainly of vegetable origin or from dairy products, and extra-virgin olive oil, and seeds, rich in alfa-linoleic acid, all of which are nutrients able to stimulate IGF-I secretion. Diet regimens characterized by too many sugars and fats should be discouraged to avoid a blunting of residual GH and IGF-I secretion. Foods rich in vitamin D and an outdoor lifestyle to increase the production at the skin level should be encouraged to take advantage of vitamin D in GH and IGF-I secretion. Foods rich in Zn, iodine, Ca^2+^, and Mg should be promoted, and attention should be paid to propose selenium supplementation to avoid its U-shaped effect on the GH/IGF-I axis.

### 7.2. Acromegaly

Nutrients can blunt both GH and IGF-I secretion. As discussed previously, a proof-of-concept study with a 2-week eucaloric very-low-carbohydrate ketogenic diet was able to decrease IGF-I or GH levels in a small cohort of patients with active disease [352], likely due to several mechanisms including decrease in insulin levels, portal circulation, ketone bodies, and FFA levels. Indeed, periodical or prolonged regimens of CR models aiming to restrict the calorie intake and the anabolic drivers could be proposed in this these patients. Another possibility, likely as a break to strictly fasting regimens, is the Okinawa diet, poor in proteins and rich in carbohydrates, two players acting on decreasing GH and IGF-I secretion, or modified diets poor in BCAAs (leucine, valine, isoleucine). Treatment or supplementation with vitamin D should be personalized to avoid higher levels of the hormone and Ca^2+^, which could further contribute to the increase in IGF-I levels. Similar attention should be paid to not exceed in vitamins A, B8, B12, or Mg and selenium. Ca^2+^ and phosphate intake should be tailored at the average level of requirement, mainly promoting unprocessed foods frequently too rich in phosphates.

## 8. Conclusions

As described above, GH/IGF-I axis homeostasis is strictly connected to all the diet components. Nutrients are plain modifiers of the GH/IGF-I axis. Identifying a diet with a healthy balanced mixture of nutrients also healthy for the mediated effects on GH and IGF-I is complicated by the manifold composition of energy regimens and synergistic effects of nutrients on each other and hormonal axes. Furthermore, food processes, both in experimental animal models and in human life, including home cooking and industrial processing are neglected factors because of difficulties in analyzing them, mainly in humans. Calories and nutrients modulate the pathways that have evolved to switch from growth and reproduction toward survival and maintenance during CR and famine [378,379]. The imbalance of one or more macro- and micronutrients could affect the short- and long-term GH and IGF-I secretion and actions, with repercussions on growth, anabolism, and nutrients sensing [380]. All the evidence discussed in this review derives from models aiming to dissect the effect of single nutrients on GH and IGF-I. However, nutritional deficiencies due to peculiar food restrictions could hide multiple deficiencies or excesses, leading to debatable results. Recently, advances in nutritional geometry have helped to study nutrition as a framework to begin deciphering how nutrients interact and orchestrate mechanisms linked to healthy aging and lifespan [381,382]. Expanding this approach to hormonal regulation, like that of the GH/IGF-I axis could add new depth and open perspectives in nutrition management, prevention, and treatment of GH/IGF-I deficiency or excess at different time points of life.

## Figures and Tables

**Table 1:** Regulation among the GH/IGF-I axis and macronutrients ↑ = increase; ↓ = decrease.

Macronutrient	Effects on GH/IGF-I Axis	Regulation by GH/IGF-I
*Carbohydrates*	Oral glucose administration: ↓ GH (↑ hypothalamic somatostatin) [106,110,111,112] ↓ ghrelin and GH after oral glucose [114] Hypoglycemia: ↑ GH [107]	GH ↓ glucose and proteins utilization and ↑ lipid utilization [1] GH ↑ insulin resistance: ↓ insulin-mediated glucose uptake in muscle; modulates insulin signaling (PI3K activity; ↑ SOCS1, SOC3) [1,99,102,103,104] IGF-I ↑ glucose uptake, ↓ gluconeogenesis and ↓ glycogenolysis [101] GH ↑ hepatic autophagy and gluconeogenesis under fasted and fat-depleted conditions [74,89,91]
*Fibers*	↑ GH and IGF-I [128] Mechanisms unexplored	
*Proteins*	Intravenous administration of AAs: ↑ GH [133,134] Oral administration of AAs: ↑ GH (mainly in females and youngers) [139,140,141,142,145,146] Basic AAs ↑ GH; leucine and valine less potent; isoleucine ↔ GH [134] Arginine ↑ GH (↓ somatostatin at hypothalamus) [135] Dietary tryptophan and methionine restriction: ↓ GH and IGF-I [173] Total protein: ↑ GH [147,148] Diets high in protein: ↑ GH and IGF-I [148,152] Formula milk with tryptophan and phenylalanine: ↑ IGF-I [151] High milk and dairy protein intake: ↑ IGF-I [155,156,157,158,159] Vegan *vs* meat-eaters and vegetarians: ↓ IGF-I and ↑ IGFBP-1/2 [153] CR and protein restriction: ↓ IGF-I [167] BCAAs: ↑ IGF-I [178]	GH/IGF-I ↓ protein breakdown and ↑ protein synthesis in muscle and other tissues [1] GH ↓ AA degradation/ureagenesis in liver [1] GH ↓ BCAAs (↑ LAT1 and uptake by muscle, ↑ gluconeogenesis) [180,181,182] IGF-I ↑ BCAAs [182]
*Lipids*	Infusion of mixed FFAs: ↓ spontaneous and GHRH-stimulated GH (by somatotropic cells or ↓ somatostatin) [52,63,191,194,195,196] Lipolysis inhibiting drugs ↑ basal and GHRH-stimulated GH [191] Diets rich in alfa-linoleic acid: ↑ IGF-I, GHR, and IGFBPs mRNA in liver; ↑ IGF-I secretion [203] Sodium salts of valerate, hexanoic, caprylic, nonanoic, and dodecanoic acids ↑ GH and PRL [204] Acetate, propionate, and butyrate ↓ GHRH, GH, PRL gene transcription in dairy cow anterior pituitary cells [207]	GH ↑ lipolysis, lipid oxidation and ketogenesis [1,185] GH regulates VLDL metabolism and availability of FFAs for peripheral tissues [188,189] GH ↑ LDL- receptor expression and HDL-cholesterol production [188,189] IGF-I ↓ plasma FFAs (↓ lipolysis) [202]

**Table 2:** Regulation among the GH-IGF-I axis, vitamins, and minerals ↑ = increase; ↓ = decrease; ↔ = no changes.

Micronutrient	Effects on GH/IGF-I Axis	Regulation by GH/IGF-I
*Vitamin D*	↑ GH (binding to pituitary VDR) [210,211] ↑ Liver IGF-I and IGFBP-3 (directly; ↑ GH; ↑ Ca^2+^ absorption in the gut) [212,216] ↑ IGF-I in chondrocytes [215]	IGF-I: ↑ 1,25-(OH)2D3 and ↑ 24,25-(OH)2D3 (kidney, placenta) [212,218,219]
*Vitamin A*	Modulates GH gene (interaction with RXR-α in the pituitary) [220] ↑ GH (non-transcriptional effect) [222] Vitamin-A-deficient rats: ↓ GH-regulated CYP2C11, CYP4A2, IGF-I, and GH-responsiveness of the JAK-STAT system [223]	
*Vitamin E*	Low levels ↑ oxidative stress in GHD children [226] No mechanistic studies	
*Vitamin B6*	Pyridoxal phosphate ↓ GH in acromegaly and infants [227] ↓ Cell proliferation and GH secretion [227]	
*Vitamin B8*	Dietary restriction in mice: ↓ IGF-I availability; ↔ GH [228]	
*Vitamin B12*	Deficiency: ↓ liver taurine; ↓ GH/IGF-I axis with GH resistance [230]	GH ↑ vitamin B12 availability [229]
*Sodium, potassium, and water*	Dehydration ↑ GH [231] Potassium depletion in animal models: ↑ intrarenal IGF-I levels, ↓ circulating and hepatic IGF-I levels [232,237,247]	IGF-I: modulation of renin release, ↓ ANP, ↑ distal tubular Na channels [232,233] GH: ↑ salt and water retention (renal tubuli) [231] GH and IGF-I: ↑ transepithelial Na transport (ENaC, cortical collecting duct) [231] GH: ↓ urinary potassium excretion [232,237,247]
*Calcium*	Basal and stimulated GH secretion depend on Ca^2+^ influx in pituitary cells through L-type Ca^2+^ channels [239,240] Pituitary extracellular Ca^2+^-sensing ↑ GH response to GHRH [242] ↑ PTH and Ca^2+^: ↓ stimulated GH secretion, ↓ IGF-I levels; tune U-shaped regulation [112,241]	GH/IGF-I: ↑ Ca^2+^ gut absorption by TRPV6 [231,244] IGF-I: ↑ Ca^2+^ reabsorption by ↑ TRPV5 in distal tubule (↑1,25-(OH)2D3) [231,245] IGF-I: absorptive hypercalciuria and ↑ fasting plasma Ca^2+^ in acromegalic patients (↑1,25-(OH)2D3) [231,246]
*Phosphorus*		GH ↑ renal phosphate absorption via Na-phosphate cotransport [249,250,253] IGF-I ↓ renal phosphate excretion via Na-phosphate cotransport [251,252,253] GH treatment and acromegaly: ↑ serum phosphate (↑ Klotho secretion in kidney, FGF23 resistance) [250,254,255]
*Magnesium*	Modulates hormone-sensitive adenylate cyclase system in somatotroph cells [256] Nutritional deficiency: ↓ IGF-I; ↔ GH secretion (GH-R or post-receptor defects) [257]	GH ↓ renal Mg wasting induced by metabolic acidosis [258,259]
*Zinc*	Regulates GH signaling ability (↑ GH more stable dimeric form) [262,263] Prolongs GH binding to GHR and ↑ GH aggregation in secretory granules [263,264,265] Main determinant of IGF-I synthesis [260,261] ↑ IGF-I-anabolic effect in osteoblastic cells [261,271,273] Deficiency: ↓ body weight, IGF-I, GH and GHBP, hepatic GHR and IGF-I expression in rats [268,269] Supplementation: ↑ GH, IGF-I, IGFBP-3 levels in animal models, GHD and healthy children [260,269,270,271,272]	
*Iron*		Hemopexin is a GH-inducible gene [275] IGFBP-3 binds transferrin [276] GH treatment ↓ ferritin and ↑ serum transferrin [280] IGF-I ↑ erythropoiesis (precursors and mature erythrocytes) [277,278]
*Iodine*	Thyroid hormones important for normal GH expression and feedback mechanisms on GHR, influencing IGF-I levels [282,283] Iodine supplementation ↑ IGF-I [282]	
*Selenium*	↓ GH and IGF-I system; U-shaped regulation [286,287,288,289] Deficiency: growth retardation; ↓ pituitary GH; ↓ IGF-I secretion/circulating levels (direct; inflammation) [290,291] ↓ Type II deiodinase selenoenzyme activity (pituitary) [290,291]	
*Manganese*	Deficiency in animals: ↓ body weight and growth, bone abnormalities, ↓ IGF-I and insulin, ↑ GH and IGFBP-3 [296,297] Mn^2+^ bioavailability could affect IGF-I and insulin signaling [298] ↑ IGF-I gene expression and IGF-IR protein expression in medial basal hypothalamus [299]	
*Copper*	Deficiency: ↓ serum IGF-I; ↑ IGF-I in bones [301] Supplementation: ↑ IGF-I and IGFBP-3 (culture media of chondrocytes) [289] ↑ GHRH, GH, and ghrelin secretion; ↓ somatostatin expression [300,302]	
*Chromium*	Negligible or quite insignificant effects [303]	

## Data Availability

Not applicable.

## References

[1] Moøller N., Joørgensen J.O.L. (2009). Effects of growth hormone on glucose, lipid, and protein metabolism in human subjects. Endocr. Rev..

[2] Bartke A., Darcy J. (2017). GH and ageing: Pitfalls and new insights. Best Pract. Res. Clin. Endocrinol. Metab..

[3] Poudel S.B., Dixit M., Neginskaya M., Nagaraj K., Pavlov E., Werner H., Yakar S. (2020). Effects of GH/IGF on the Aging Mitochondria. Cells.

[4] Donohoe C.L., Lysaght J., O’Sullivan J., Reynolds J.V. (2017). Emerging Concepts Linking Obesity with the Hallmarks of Cancer. Trends Endocrinol. Metab..

[5] López-Otín C., Kroemer G. (2021). Hallmarks of Health. Cell.

[6] Chen E.Y., Liao Y.C., Smith D.H., Barrera-Saldaña H.A., Gelinas R.E., Seeburg P.H. (1989). The human growth hormone locus: Nucleotide sequence, biology, and evolution. Genomics.

[7] Baumann G.P. (2009). Growth hormone isoforms. Growth Horm. IGF Res..

[8] Ho K.Y., Veldhuis J.D., Johnson M.L., Furlanetto R., Evans W.S., Alberti K.G.M.M., Thorner M.O. (1988). Fasting enhances growth hormone secretion and amplifies the complex rhythms of growth hormone secretion in man. J. Clin. Investig..

[9] Hartman M.L., Veldhuis J.D., Thorner M.O. (1993). Normal control of growth hormone secretion. Horm. Res..

[10] Bonert V., Melmed S. (2017). The Pituitary.

[11] Giustina A., Veldhuis J.D. (1998). Pathophysiology of the Neuroregulation of Growth Hormone Secretion in Experimental Animals and the Human. Endocr. Rev..

[12] Eigler T., Ben-Shlomo A. (2014). Somatostatin system: Molecular mechanisms regulating anterior pituitary hormones. J. Mol. Endocrinol..

[13] Ghigo E., Arvat E., Bellone J., Ramunni J., Camanni F. (1993). Neurotransmitter Control of Growth Hormone Secretion in Humans. J. Pediatr. Endocrinol. Metab..

[14] Vance M.L., Hartman M.L., Thorner M.O. (1992). Growth hormone and nutrition. Horm. Res. Paediatr..

[15] Hartman M.L., Clayton P.E., Johnson M.L., Celniker A., Perlman A.J., Alberti K.G.M.M., Thorner M.O. (1993). A low dose euglycemic infusion of recombinant human insulin-like growth factor I rapidly suppresses fasting-enhanced pulsatile growth hormone secretion in humans. J. Clin. Investig..

[16] Vottero A., Guzzetti C., Loche S. (2013). New aspects of the physiology of the GH-IGF-1 axis. Hormone Resistance and Hypersensitivity: From Genetics to Clinical Management.

[17] Mertani H.C., Delehaye-Zervas M.C., Martini J.F., Postel-Vinay M.C., Morel G. (1995). Localization of growth hormone receptor messenger RNA in human tissues. Endocrine.

[18] Dehkhoda F., Lee C.M.M., Medina J., Brooks A.J. (2018). The growth hormone receptor: Mechanism of receptor activation, cell signaling, and physiological aspects. Front. Endocrinol..

[19] Lanning N.J., Carter-Su C. (2006). Recent advances in growth hormone signaling. Rev. Endocr. Metab. Disord..

[20] Carter-Su C., Schwartz J., Argetsinger L.S. (2016). Growth hormone signaling pathways. Growth Horm. IGF Res..

[21] Kaplan S.A., Cohen P. (2007). Review: The somatomedin hypothesis 2007: 50 Years later. J. Clin. Endocrinol. Metab..

[22] Siddle K. (2011). Signalling by insulin and IGF receptors: Supporting acts and new players. J. Mol. Endocrinol..

[23] Messina J. (2010). Insulin as a growth-promoting hormone. Comprehensive Physiology.

[24] Hong S., Mannan A.M., Inoki K. (2012). Evaluation of the nutrient-sensing mTOR pathway. Methods Mol. Biol..

[25] Forbes B.E., Blyth A.J., Wit J.M. (2020). Disorders of IGFs and IGF-1R signaling pathways. Mol. Cell. Endocrinol..

[26] Rajaram S., Baylink D.J., Mohan S. (1997). Insulin-Like Growth Factor-Binding Proteins in Serum and Other Biological Fluids: Regulation and Functions. Endocr. Rev..

[27] Rosenzweig S.A. (2004). What’s new in the IGF-binding proteins?. Growth Horm. IGF Res..

[28] Blum W.F., Albertsson-Wikland K., Rosberg S., Ranke M.B. (1993). Serum levels of insulin-like growth factor I (IGF-I) and IGF binding protein 3 reflect spontaneous growth hormone secretion. J. Clin. Endocrinol. Metab..

[29] Maures T.J., Duan C. (2002). Structure, developmental expression, and physiological regulation of zebrafish IGF binding protein-1. Endocrinology.

[30] Kajimura S., Aida K., Duan C. (2006). Understanding Hypoxia-Induced Gene Expression in Early Development: In Vitro and In Vivo Analysis of Hypoxia-Inducible Factor 1-Regulated Zebra Fish Insulin-Like Growth Factor Binding Protein 1 Gene Expression. Mol. Cell. Biol..

[31] O’Brien R.M., Noisin E.L., Suwanichkul A., Yamasaki T., Lucas P.C., Wang J.C., Powell D.R., Granner D.K. (1995). Hepatic nuclear factor 3- and hormone-regulated expression of the phosphoenolpyruvate carboxykinase and insulin-like growth factor-binding protein 1 genes. Mol. Cell. Biol..

[32] Veldhuis J.D., Johnson M.L. (1986). Cluster analysis: A simple, versatile, and robust algorithm for endocrine pulse detection. Am. J. Physiol. Endocrinol. Metab..

[33] Veldhuis J.D., Carlson M.L., Johnson M.L. (1987). The pituitary gland secretes in bursts: Appraising the nature of glandular secretory impulses by simultaneous multiple-parameter deconvolution of plasma hormone concentrations. Proc. Natl. Acad. Sci. USA.

[34] Hartman M.L., Veldhuis J.D., Johnson M.L., Lee M.M., Alberti K.G.M.M., Samojlik E., Thorner M.O. (1992). Augmented growth hormone (GH) secretory burst frequency and amplitude mediate enhanced GH secretion during a two-day fast in normal men. J. Clin. Endocrinol. Metab..

[35] Riedel M., Hoeft B., Blum W.F., von zur Mühlen A., Brabant G. (1995). Pulsatile growth hormone secretion in normal-weight and obese men: Differential metabolic regulation during energy restriction. Metabolism.

[36] Sakharova A.A., Horowitz J.F., Surya S., Goldenberg N., Harber M.P., Symons K., Barkan A. (2008). Role of growth hormone in regulating lipolysis, proteolysis, and hepatic glucose production during fasting. J. Clin. Endocrinol. Metab..

[37] Clemmons D.R., Klibanski A., Underwood L.E., McArthur J.W., Ridgway E.C., Beitins I.Z., Van Wyk J.J. (1981). Reduction of plasma immunoreactive somatomedin C during fasting in humans. J. Clin. Endocrinol. Metab..

[38] Merimee T.J., Zapf J., Froesch E.R. (1982). Insulin-Like Growth Factors in the Fed and Fasted States. J. Clin. Endocrinol. Metab..

[39] Underwood L.E., Thissen E.P., Ketelslegers J.M. (1994). Nutritional regulation of the insulin-like growth factors. Endocr. Rev..

[40] Counts D.R., Gwirtsman H., Carlsson L.M.S., Lesem M. (1992). The effect of anorexia nervosa and refeeding on growth hormone-binding protein, the insulin-like growth factors (IGFs), and the IGF-binding proteins. J. Clin. Endocrinol. Metab..

[41] Argente J., Caballo N., Barrios V., Muñoz M.T., Pozo J., Chowen J.A., Morandé G., Hernández M. (1997). Multiple Endocrine Abnormalities of the Growth Hormone and Insulin-Like Growth Factor Axis in Patients with Anorexia Nervosa: Effect of Short- and Long-Term Weight Recuperation 1. J. Clin. Endocrinol. Metab..

[42] Fazeli P.K., Klibanski A. (2014). Determinants of GH resistance in malnutrition. J. Endocrinol..

[43] Clemmons D.R. (2012). Metabolic Actions of Insulin-Like Growth Factor-I in Normal Physiology and Diabetes. Endocrinol. Metab. Clin. N. Am..

[44] Baxter R.C., Bryson J.M., Turtle J.R. (1981). The effect of fasting on liver receptors for prolactin and growth hormone. Metabolism.

[45] Straus D.S., Takemoto C.D. (1990). Effect of fasting on insulin-like growth factor-I (IGF-I) and growth hormone receptor mRNA levels and IGF-I gene transcription in rat liver. Mol. Endocrinol..

[46] Huang Z., Huang L., Waters M.J., Chen C. (2020). Insulin and Growth Hormone Balance: Implications for Obesity. Trends Endocrinol. Metab..

[47] Aimaretti G., Colao A., Corneli G., Pivonello R., Maccario M., Morrison K., Pflaum C.D., Strasburger C.J., Lombardi G., Ghigo E. (1999). The study of spontaneous GH secretion after 36-h fasting distinguishes between GH-deficient and normal adults. Clin. Endocrinol. (Oxf.).

[48] Grottoli S., Gasco V., Mainolfi A., Beccuti G., Corneli G., Aimaretti G., Dieguez C., Casanueva F., Ghigo E. (2008). Growth hormone/insulin-like growth factor I axis, glucose metabolism, and lypolisis but not leptin show some degree of refractoriness to short-term fasting in acromegaly. J. Endocrinol. Investig..

[49] Hartman M.L., Thorner M.O. Fasting-induced enhancement of pulsatile growth hormone (GH) secretion is rapidly abolished by refeeding. Proceedings of the 72nd Meeting of the Endocrine Society.

[50] Cornford A.S., Barkan A.L., Horowitz J.F. (2011). Rapid suppression of growth hormone concentration by overeating: Potential mediation by hyperinsulinemia. J. Clin. Endocrinol. Metab..

[51] Briard N., Rico-Gomez M., Guillaume V., Sauze N., Vuaroqueaux V., Dadoun F., Le Bouc Y., Oliver C., Dutour A. (1998). Hypothalamic mediated action of free fatty acid on growth hormone secretion in sheep. Endocrinology.

[52] Casanueva F.F., Villanueva L., Dieguez C., Diaz Y., Cabranes J.A., Szoke B., Scanlon M.F., Schally A.V., Fernandez-Cruz A. (1987). Free fatty acids block growth hormone (GH) releasing hormone-stimulated gh secretion in man directly at the pituitary. J. Clin. Endocrinol. Metab..

[53] Bang P., Brismar K., Rosenfeld R.G., Hall K. (1994). Fasting affects serum insulin-like growth factors (IGFs) and IGF-binding proteins differently in patients with noninsulin-dependent diabetes mellitus versus healthy nonobese and obese subjects. J. Clin. Endocrinol. Metab..

[54] Frystyk J., Delhanty P.J.D., Skjærbæk C., Baxter R.C. (1999). Changes in the circulating IGF system during short-term fasting and refeeding in rats. Am. J. Physiol. Endocrinol. Metab..

[55] Maccario M., Aimaretti G., Grottoli S., Gauna C., Tassone F., Corneli G., Rossetto R., Wu Z., Strasburger C.J., Ghigo E. (2001). Effects of 36 hour fasting on GH/IGF-I axis and metabolic parameters in patients with simple obesity. Comparison with normal subjects and hypopituitary patients with severe GH deficiency. Int. J. Obes..

[56] Luque R.M., Kineman R.D. (2006). Impact of obesity on the growth hormone axis: Evidence for a direct inhibitory effect of hyperinsulinemia on pituitary function. Endocrinology.

[57] Gahete M.D., Córdoba-Chaćon J., Lin Q., Brüning J.C., Kahn C.R., Castaño J.P., Christian H., Luque R.M., Kineman R.D. (2013). Insulin and IGF-I inhibit GH synthesis and release in vitro and in vivo by separate mechanisms. Endocrinology.

[58] Gasco V., Pagano L., Prodam F., Marzullo P., Ghigo E.A.G., Clemmons D., Attanasio A. (2009). Hypothalamic-Pituitary Disease and Obesity.

[59] Berryman D.E., Glad C.A.M., List E.O., Johannsson G. (2013). The GH/IGF-1 axis in obesity: Pathophysiology and therapeutic considerations. Nat. Rev. Endocrinol..

[60] Grottoli S., Gauna C., Tassone F., Aimaretti G., Corneli G., Wu Z., Strasburger C.J., Dieguez C., Casanueva F.F., Ghigo E. (2003). Both fasting-induced leptin reduction and GH increase are blunted in Cushing’s syndrome and in simple obesity. Clin. Endocrinol. (Oxf.).

[61] Bonert V.S., Elashoff J.D., Barnett P., Melmed S. (2004). Body mass index determines evoked growth hormone (GH) responsiveness in normal healthy male subjects: Diagnostic caveat for adult GH deficiency. J. Clin. Endocrinol. Metab..

[62] Qu X.D., Gaw Gonzalo I.T., Al Sayed M.Y., Cohan P., Christenson P.D., Swerdloff R.S., Kelly D.F., Wang C. (2005). Influence of body mass index and gender on growth hormone (GH) responses to GH-releasing hormone plus arginine and insulin tolerance tests. J. Clin. Endocrinol. Metab..

[63] Williams T., Berelowitz M., Joffe S.N., Thorner M.O., Rivier J., Vale W., Frohman L.A. (1984). Impaired Growth Hormone Responses to Growth Hormone–Releasing Factor in Obesity. N. Engl. J. Med..

[64] Rasmussen M.H., Hvidberg A., Juul A., Main K.M., Gotfredsen A., Skakkebaek N.E., Hilsted J. (1995). Massive weight loss restores 24-hour growth hormone release profiles and serum insulin-like growth factor-I levels in obese subjects. J. Clin. Endocrinol. Metab..

[65] De Marinis L., Bianchi A., Mancini A., Gentilella R., Perrelli M., Giampietro A., Porcelli T., Tilaro L., Fusco A., Valle D. (2004). Growth Hormone Secretion and Leptin in Morbid Obesity before and after Biliopancreatic Diversion: Relationships with Insulin and Body Composition. J. Clin. Endocrinol. Metab..

[66] Yang J., Brown M.S., Liang G., Grishin N.V., Goldstein J.L. (2008). Identification of the Acyltransferase that Octanoylates Ghrelin, an Appetite-Stimulating Peptide Hormone. Cell.

[67] Devesa J. (2021). The Complex World of Regulation of Pituitary Growth Hormone Secretion: The Role of Ghrelin, Klotho, and Nesfatins in It. Front. Endocrinol. (Lausanne).

[68] Devesa J., Lima L., Tresguerres J.A.F. (1992). Neuroendocrine control of growth hormone secretion in humans. Trends Endocrinol. Metab..

[69] Root A.W., Root M.J. (2002). Clinical pharmacology of human growth hormone and its secretagogues. Curr. Drug Targets. Immune. Endocr. Metabol. Disord..

[70] Nakazato M., Murakami N., Date Y., Kojima M., Matsuo H., Kangawa K., Matsukura S. (2001). A role for ghrelin in the central regulation of feeding. Nature.

[71] Kojima M., Kangawa K. (2005). Ghrelin: Structure and function. Physiol. Rev..

[72] Malik S., McGlone F., Bedrossian D., Dagher A. (2008). Ghrelin Modulates Brain Activity in Areas that Control Appetitive Behavior. Cell Metab..

[73] Kanoski S.E., Fortin S.M., Ricks K.M., Grill H.J. (2013). Ghrelin signaling in the ventral hippocampus stimulates learned and motivational aspects of feeding via PI3K-Akt signaling. Biol. Psychiatry.

[74] Yanagi S., Sato T., Kangawa K., Nakazato M. (2018). The Homeostatic Force of Ghrelin. Cell Metab..

[75] Gortan Cappellari G., Barazzoni R. (2019). Ghrelin forms in the modulation of energy balance and metabolism. Eat. Weight Disord..

[76] Romere C., Duerrschmid C., Bournat J., Constable P., Jain M., Xia F., Saha P.K., Del Solar M., Zhu B., York B. (2016). Asprosin, a Fasting-Induced Glucogenic Protein Hormone. Cell.

[77] Duerrschmid C., He Y., Wang C., Li C., Bournat J.C., Romere C., Saha P.K., Lee M.E., Phillips K.J., Jain M. (2017). Asprosin is a centrally acting orexigenic hormone. Nat. Med..

[78] Prodam F., Me E., Riganti F., Gramaglia E., Bellone S., Baldelli R., Rapa A., Van Der Lely A.J., Bona G., Ghigo E. (2006). The nutritional control of ghrelin secretion in humans: The effects of enteral vs. parenteral nutrition. Eur. J. Nutr..

[79] Prodam F., Monzani A., Ricotti R., Marolda A., Bellone S., Aimaretti G., Roccio M., Bona G. (2014). Systematic review of ghrelin response to food intake in pediatric age, from neonates to adolescents. J. Clin. Endocrinol. Metab..

[80] Müller T.D., Nogueiras R., Andermann M.L., Andrews Z.B., Anker S.D., Argente J., Batterham R.L., Benoit S.C., Bowers C.Y., Broglio F. (2015). Ghrelin. Mol. Metab..

[81] Muller A.F., Lamberts S.W.J., Janssen J.A.M.J.L., Hofland L.J., Van Koetsveld P., Bidlingmaier M., Strasburger C.J., Ghigo E., Van der Lely A.J. (2002). Ghrelin drives GH secretion during fasting in man. Eur. J. Endocrinol..

[82] Koutkia P., Canavan B., Breu J., Johnson M.L., Grinspoon S.K. (2004). Nocturnal ghrelin pulsatility and response to growth hormone secretagogues in healthy men. Am. J. Physiol. Endocrinol. Metab..

[83] Nass R., Farhy L.S., Liu J., Prudom C.E., Johnson M.L., Veldhuis P., Pezzoli S.S., Oliveri M.C., Gaylinn B.D., Geysen H.M. (2008). Evidence for acyl-ghrelin modulation of growth hormone release in the fed state. J. Clin. Endocrinol. Metab..

[84] Broglio F., Prodam F., Riganti F., Gottero C., Destefanis S., Granata R., Muccioli G., Abribat T., van der Lely A.J., Ghigo E. (2008). The continuous infusion of acylated ghrelin enhances growth hormone secretion and worsens glucose metabolism in humans. J. Endocrinol. Investig..

[85] Espelund U., Hansen T.K., Højlund K., Beck-Nielsen H., Clausen J.T., Hansen B.S., Ørskov H., Jørgensen J.O.L., Frystyk J. (2005). Fasting unmasks a strong inverse association between Ghrelin and Cortisol in serum: Studies in obese and normal-weight subjects. J. Clin. Endocrinol. Metab..

[86] Nørrelund H., Hansen T.K., Ørskov H., Hosoda H., Kojima M., Kangawa K., Weeke J., Møller N., Christiansen J.S., Jørgensen J.O.L. (2002). Ghrelin immunoreactivity in human plasma is suppressed by somatostatin. Clin. Endocrinol. (Oxf.).

[87] Natalucci G., Riedl S., Gleiss A., Zidek T., Frisch H. (2005). Spontaneous 24-h ghrelin secretion pattern in fasting subjects: Maintenance of a meal-related pattern. Eur. J. Endocrinol..

[88] Avram A.M., Jaffe C.A., Symons K.V., Barkan A.L. (2005). Endogenous circulating ghrelin does not mediate growth hormone rhythmicity or response to fasting. J. Clin. Endocrinol. Metab..

[89] Zhao T.J., Liang G., Li R.L., Xie X., Sleeman M.W., Murphy A.J., Valenzuela D.M., Yancopoulos G.D., Goldstein J.L., Brown M.S. (2010). Ghrelin O-acyltransferase (GOAT) is essential for growth hormone-mediated survival of calorie-restricted mice. Proc. Natl. Acad. Sci. USA.

[90] Goldstein J.L., Zhao T.J., Li R.L., Sherbet D.P., Liang G., Brown M.S. (2011). Surviving starvation: Essential role of the ghrelin-growth hormone axis. Cold Spring Harb. Symp. Quant. Biol..

[91] Zhang Y., Fang F., Goldstein J.L., Brown M.S., Zhao T.J. (2015). Reduced autophagy in livers of fasted, fat-depleted, ghrelin-deficient mice: Reversal by growth hormone. Proc. Natl. Acad. Sci. USA.

[92] Ge X., Yang H., Bednarek M.A., Galon-Tilleman H., Chen P., Chen M., Lichtman J.S., Wang Y., Dalmas O., Yin Y. (2018). LEAP2 Is an Endogenous Antagonist of the Ghrelin Receptor. Cell Metab..

[93] Mani B.K., Puzziferri N., He Z., Rodriguez J.A., Osborne-Lawrence S., Metzger N.P., Chhina N., Gaylinn B., Thorner M.O., Louise Thomas E. (2019). LEAP2 changes with body mass and food intake in humans and mice. J. Clin. Investig..

[94] Nelson D.L., Cox M.M. (2017). Lehninger Principles of Biochemistry.

[95] SINU—Società Italiana di Nutrizione Umana. https://sinu.it/.

[96] Trumbo P., Schlicker S., Yates A.A., Poos M. (2002). Dietary reference intakes for energy, carbohydrate, fiber, fat, fatty acids, cholesterol, protein and amino acids. J. Am. Diet. Assoc..

[97] Dietary Guidelines for Americans, 2020–2025 and Online Materials|Dietary Guidelines for Americans. https://www.dietaryguidelines.gov/resources/2020-2025-dietary-guidelines-online-materials.

[98] Dominici F.P., Turyn D. (2002). Growth hormone-induced alterations in the insulin-signaling system. Exp. Biol. Med..

[99] Oliveira C.R.P., Meneguz-Moreno R.A., Aguiar-Oliveira M.H., Barreto-Filho J.A.S. (2011). Emerging role of the GH/IGF-I on cardiometabolic control. Arq. Bras. Cardiol..

[100] Chiarelli F., Giannini C., Mohn A. (2004). Growth, growth factors and diabetes. Eur. J. Endocrinol..

[101] LeRoith D., Yakar S. (2007). Mechanisms of disease: Metabolic effects of growth hormone and insulin-like growth factor 1. Nat. Clin. Pract. Endocrinol. Metab..

[102] Lebrun P., Van Obberghen E. (2008). SOCS proteins causing trouble in insulin action. Acta Physiol. (Oxf.).

[103] Del Rincon J.P., Iida K., Gaylinn B.D., McCurdy C.E., Leitner J.W., Barbour L.A., Kopchick J.J., Friedman J.E., Draznin B., Thorner M.O. (2007). Growth hormone regulation of p85α expression and phosphoinositide 3-kinase activity in adipose tissue: Mechanism for growth hormone-mediated insulin resistance. Diabetes.

[104] Barbour L.A., Rahman S.M., Gurevich I., Leitner J.W., Fischer S.J., Roper M.D., Knotts T.A., Vo Y., McCurdy C.E., Yakar S. (2005). Increased P85α is a potent negative regulator of skeletal muscle insulin signaling and induces in vivo insulin resistance associated with growth hormone excess. J. Biol. Chem..

[105] Yuen K.C.J., Frystyk J., White D.K., Twickler T.B., Koppeschaar H.P.F., Harris P.E., Fryklund L., Murgatroyd P.R., Dunger D.B. (2005). Improvement in insulin sensitivity without concomitant changes in body composition and cardiovascular risk markers following fixed administration of a very low growth hormone (GH) dose in adults with severe GH deficiency. Clin. Endocrinol. (Oxf.).

[106] Hunter W.M., Willoughby J.M., Strong J.A. (1968). Plasma insulin and growth hormone during 22-hour fasts and after graded glucose loads in six healthy adults. J. Endocrinol..

[107] Roth J., Glick S.M., Yalow R.S., Berson S.A. (1963). Hypoglycemia: A potent stimulus to secretion of growth hormone. Science.

[108] Roth J., Glick S., Yalow R., Berson S. (1963). Secretion of human growth hormone: Physiologic and experimental modification. Metabolism.

[109] Yalow R.S., Goldsmith S.J., Berson S.A. (1969). Influence of physiologic fluctuations in plasma growth hormone on glucose tolerance. Diabetes.

[110] Penalva A., Burguera B., Cusahie X., Tresguerres J.A.F., Dieguez C., Casanueva F.F. (1989). Activation of cholinergic neurotransmission by pyridostigmine reverses the inhibitory effect of hyperglycemia on growth hormone (GH) releasing hormone-induced GH secretion in man: Does acute hyperglycemia act through hypothalamic release of somatostatin?. Neuroendocrinology.

[111] Masuda A., Shibasaki T., Nakahara M., Imaki T., Kiyosawa Y., Jibiki K., Demura H., Shizume K., Ling N. (1985). The effect of glucose on growth hormone (gh)-releasing hormone-mediated gh secretion in man. J. Clin. Endocrinol. Metab..

[112] Gasperi M., Cecconi E., Grasso L., Bartalena L., Centoni R., Aimaretti G., Broglio F., Miccoli P., Marcocci C., Ghigo E. (2002). GH secretion is impaired in patients with primary hyperparathyroidism. J. Clin. Endocrinol. Metab..

[113] Friend K., Iranmanesh A., Login I.S., Veldhuis J.D. (1997). Pyridostigmine treatment selectively amplifies the mass of GH secreted per burst without altering GH burst frequency, half-life, basal GH secretion or the orderliness of GH release. Eur. J. Endocrinol..

[114] Nakagawa E., Nagaya N., Okumura H., Enomoto M., Oya H., Ono F., Hosoda H., Kojima M., Kangawa K. (2002). Hyperglycaemia suppresses the secretion of ghrelin, a novel growth-hormone-releasing peptide: Responses to the intravenous and oral administration of glucose. Clin. Sci..

[115] Shiiya T., Nakazato M., Mizuta M., Date Y., Mondal M.S., Tanaka M., Nozoe S.I., Hosoda H., Kangawa K., Matsukura S. (2002). Plasma ghrelin levels in lean and obese humans and the effect of glucose on ghrelin secretion. J. Clin. Endocrinol. Metab..

[116] Teff K.L., Elliott S.S., Tschöp M., Kieffer T.J., Rader D., Heiman M., Townsend R.R., Keim N.L., D’Alessio D., Havel P.J. (2004). Dietary fructose reduces circulating insulin and leptin, attenuates postprandial suppression of ghrelin, and increases triglycerides in women. J. Clin. Endocrinol. Metab..

[117] Pena-Bello L., Pertega-Diaz S., Outeiriño-Blanco E., Garcia-Buela J., Tovar S., Sangiao-Alvarellos S., Dieguez C., Cordido F. (2015). Effect of oral glucose administration on rebound growth hormone release in normal and obese women: The role of adiposity, insulin sensitivity and ghrelin. PLoS ONE.

[118] Gottero C., Bellone S., Rapa A., van Koetsveld P., Vivenza D., Prodam F., Benso A., Destefanis S., Gauna C., Bellone J. (2003). Standard light breakfast inhibits circulating ghrelin level to the same extent of oral glucose load in humans, despite different impact on glucose and insulin levels. J. Endocrinol. Investig..

[119] Lucidi P., Murdolo G., Di Loreto C., De Cicco A., Parlanti N., Fanelli C., Santeusanio F., Bolli G.B., De Feo P. (2002). Ghrelin is not necessary for adequate hormonal counterregulation of insulin-induced hypoglycemia. Diabetes.

[120] McCowen K.C., Maykel J.A., Bistrian B.R., Ling P.R. (2002). Circulating ghrelin concentrations are lowered by intravenous glucose or hyperinsulinemic euglycemic conditions in rodents. J. Endocrinol..

[121] Foster-Schubert K.E., Overduin J., Prudom C.E., Liu J., Callahan H.S., Gaylinn B.D., Thorner M.O., Cummings D.E. (2008). Acyl and total ghrelin are suppressed strongly by ingested proteins, weakly by lipids, and biphasically by carbohydrates. J. Clin. Endocrinol. Metab..

[122] Broglio F., Arvat E., Benso A., Gottero C., Muccioli G., Papotti M., van der Lely A.J., Deghenghi R., Ghigo E. (2001). Ghrelin, a Natural GH Secretagogue Produced by the Stomach, Induces Hyperglycemia and Reduces Insulin Secretion in Humans. J. Clin. Endocrinol. Metab..

[123] Slavin J. (2013). Fiber and prebiotics: Mechanisms and health benefits. Nutrients.

[124] Augustin L.S.A., Aas A.M., Astrup A., Atkinson F.S., Baer-Sinnott S., Barclay A.W., Brand-Miller J.C., Brighenti F., Bullo M., Buyken A.E. (2020). Dietary fibre consensus from the international carbohydrate quality consortium (Icqc). Nutrients.

[125] Tungland B.C., Meyer D. (2002). Nondigestible oligo-and polysaccharides (dietary fiber): Their physiology and role in human health and food. Compr. Rev. Food Sci. Food Saf..

[126] Cani P.D. (2019). Microbiota and metabolites in metabolic diseases. Nat. Rev. Endocrinol..

[127] Cani P.D. (2019). Targeting gut microbiota with a complex mix of dietary fibers improves metabolic diseases. Kidney Int..

[128] Denny-Brown S., Stanley T.L., Grinspoon S.K., Makimura H. (2012). The association of macro- and micronutrient intake with growth hormone secretion. Growth Horm. IGF Res..

[129] Lopez M., Mohiuddin S. (2021). Biochemistry, Essential Amino Acids. StatPearls.

[130] Griminger P., Scanes C.G. (1986). Protein Metabolism. Avian Physiology.

[131] World Health Organization (2000). Obesity: Preventing and Managing the Global Epidemic.

[132] USDA. https://www.usda.gov/.

[133] Chromiak J.A., Antonio J. (2002). Use of amino acids as growth hormone-releasing agents by athletes. Nutrition.

[134] Knopf R.F., Conn J.W., Fajans S.S., Floyd J.C., Guntsche E.M., Rull J.A. (1965). Plasma Growth Hormone Response to Intravenous Administration of Amino Acids. J. Clin. Endocrinol. Metab..

[135] Alba-Roth J., Müller O.A., Schopohl J., Von Werder K. (1988). Arginine stimulates growth hormone secretion by suppressing endogenous somatostatin secretion. J. Clin. Endocrinol. Metab..

[136] Ghigo E., Aimaretti G., Corneli G. (2008). Diagnosis of adult GH deficiency. Growth Horm. IGF Res..

[137] Prodam F., Pagano L., Corneli G., Golisano G., Belcastro S., Busti A., Gasco V., Beccuti G., Grottoli S., Di Somma C. (2008). Update on epidemiology, etiology, and diagnosis of adult growth hormone deficiency. J. Endocrinol. Investig..

[138] Gröschl M., Knerr I., Topf H.G., Schmid P., Rascher W., Rauh M. (2003). Endocrine responses to the oral ingestion of a physiological dose of essential amino acids in humans. J. Endocrinol..

[139] Collier S.R., Casey D.P., Kanaley J.A. (2005). Growth hormone responses to varying doses of oral arginine. Growth Horm. IGF Res..

[140] Welbourne T.C. (1995). Increased plasma bicarbonate and growth hormone after an oral glutamine load. Am. J. Clin. Nutr..

[141] Isidori A., Lo Monaco A., Cappa M. (1981). A study of growth hormone release in man after oral administration of amino acids. Curr. Med. Res. Opin..

[142] Suminski R.R., Robertson R.J., Goss F.L., Arslanian S., Kang J., DaSilva S., Utter A.C., Metz K.F. (1997). Acute effect of amino acid ingestion and resistance exercise on plasma growth hormone concentration in young men. Int. J. Sport Nutr. Exerc. Metab..

[143] Lambert M.I., Hefer J.A., Millar R.P., Macfarlane P.W. (1993). Failure of commercial oral amino acid supplements to increase serum growth hormone concentrations in male body-builders. Int. J. Sport Nutr..

[144] Hickson J.F.J., Bucci L., Pivarnik J.M., Wolinsky I., Mcmahon J.C., Turner S.D. (1990). Ornithine ingestion and growth hormone release in bodybuilders. Nutr. Res..

[145] Merimee T.J., Rabinowitz D., Fineberg S.E. (1969). Arginine-Initiated Release of Human Growth Hormone. N. Engl. J. Med..

[146] Tanaka K., Inoue S., Shiraki J., Shishido T., Saito M., Numata K., Takamura Y. (1991). Age-related decrease in plasma growth hormone: Response to growth hormone-releasing hormone, arginine, and l-dopa in obesity. Metabolism.

[147] Van Vught A.J.A.H., Nieuwenhuizen A.G., Brummer R.J.M., Westerterp-Plantenga M.S. (2008). Effects of oral ingestion of amino acids and proteins on the somatotropic axis. J. Clin. Endocrinol. Metab..

[148] Sellini M., Fierro A., Marchesi L., Manzo G., Giovannini C. (1981). Behavior of Basal Values and Circadian Rhythm of ACTH, Cortisol, PRL and GH in a High-Protein Diet. Boll. Soc. Ital. Biol. Sper..

[149] Galbo H., Christensen N.J., Mikines K.J., Sonne B., Hilsted J., Hagen C., Fahrenkrug J. (1981). The effect of fasting on the hormonal response to graded exercise. J. Clin. Endocrinol. Metab..

[150] Quirion A., Brisson G., De Carufel D., Laurencelle L., Therminarias A., Vogelaere P. (1988). Influence of exercise and dietary modifications on plasma human growth hormone, insulin and FFA. J Sport. Med. Phys. Fit..

[151] Fleddermann M., Demmelmair H., Grote V., Bidlingmaier M., Grimminger P., Bielohuby M., Koletzko B. (2017). Role of selected amino acids on plasma IGF-I concentration in infants. Eur. J. Nutr..

[152] Levine M.E., Suarez J.A., Brandhorst S., Balasubramanian P., Cheng C.W., Madia F., Fontana L., Mirisola M.G., Guevara-Aguirre J., Wan J. (2014). Low protein intake is associated with a major reduction in IGF-1, cancer, and overall mortality in the 65 and younger but not older population. Cell Metab..

[153] Allen N., Appleby P., Davey G., Kaaks R., Rinaldi S., Key T. (2002). The associations of diet with serum insulin-like growth factor I and its main binding proteins in 292 women meat-eaters, vegetarians, and vegans. Cancer Epidemiol. Biomarkers Prev..

[154] Hoppe C., Udam T.R., Lauritzen L., Mølgaard C., Juul A., Michaelsen K.F. (2004). Animal protein intake, serum insulin-like growth factor I, and growth in healthy 2.5-y-old Danish children. Am. J. Clin. Nutr..

[155] Romo Ventura E., Konigorski S., Rohrmann S., Schneider H., Stalla G.K., Pischon T., Linseisen J., Nimptsch K. (2020). Association of dietary intake of milk and dairy products with blood concentrations of insulin-like growth factor 1 (IGF-1) in Bavarian adults. Eur. J. Nutr..

[156] Crowe F.L., Key T.J., Allen N.E., Appleby P.N., Roddam A., Overvad K., Grønbæk H., Tjønneland A., Halkjær J., Dossus L. (2009). The association between diet and serum concentrations of IGF-I, IGFBP-1, IGFBP-2, and IGFBP-3 in the European prospective investigation into cancer and nutrition. Cancer Epidemiol. Biomarkers Prev..

[157] Larsson S.C., Wolk K., Brismar K., Wolk A. (2005). Association of diet with serum insulin-like growth factor I in middle-aged and elderly men. Am. J. Clin. Nutr..

[158] Norat T., Dossus L., Rinaldi S., Overvad K., Grønbæk H., Tjønneland A., Olsen A., Clavel-Chapelon F., Boutron-Ruault M.C., Boeing H. (2007). Diet, serum insulin-like growth factor-I and IGF-binding protein-3 in European women. Eur. J. Clin. Nutr..

[159] Beasley J.M., Gunter M.J., Lacroix A.Z., Prentice R.L., Neuhouser M.L., Tinker L.F., Vitolins M.Z., Strickler H.D. (2014). Associations of serum insulin-like growth factor-I and insulin-like growth factor-binding protein 3 levels with biomarker-calibrated protein, dairy product and milk intake in the Women’s Health Initiative. Br. J. Nutr..

[160] Takahashi S., Kajikawa M., Umezawa T., Takahashi S., Kato H., Miura Y., Nam T., Noguchi T., Naito H. (1990). Effect of dietary proteins on the plasma immunoreactive insulin-like growth factor-1/somatomedin C concentration in the rat. Br. J. Nutr..

[161] Takenaka A., Oki N., Takahashi S.I., Noguchi T. (2000). Dietary restriction of single essential amino acids reduces plasma insulin-like growth factor-I (IGF-I) but does not affect plasma IGF-binding protein-1 in rats. J. Nutr..

[162] Thissen J.P., Pucilowska J.B., Underwood L.E. (1994). Differential regulation of insulin-like growth factor I (IGF-I) and IGF Binding Protein-1 messenger ribonucleic acids by amino acid availability and growth hormone in rat hepatocyte primary culture. Endocrinology.

[163] Philipps A.F., Dvořák B., Kling P.J., Grille J.G., Koldovský O. (2000). Absorption of Milk-Borne Insulin-Like Growth Factor-I into Portal Blood of Suckling Rats. J. Pediatr. Gastroenterol. Nutr..

[164] Fürstenberger G., Senn H.J. (2002). Insulin-like growth factors and cancer. Lancet Oncol..

[165] Keller U. (2019). Nutritional Laboratory Markers in Malnutrition. J. Clin. Med..

[166] Paszynska E., Dmitrzak-Weglarz M., Slopien A., Tyszkiewicz-Nwafor M., Rajewski A. (2016). Salivary and serum insulin-like growth factor (IGF-1) assays in anorexic patients. World J. Biol. Psychiatry.

[167] Fontana L., Partridge L. (2015). Promoting health and longevity through diet: From model organisms to humans. Cell.

[168] Brandhorst S., Choi I.Y., Wei M., Cheng C.W., Sedrakyan S., Navarrete G., Dubeau L., Yap L.P., Park R., Vinciguerra M. (2015). A Periodic Diet that Mimics Fasting Promotes Multi-System Regeneration, Enhanced Cognitive Performance, and Healthspan. Cell Metab..

[169] Tremblay F., Lavigne C., Jacques H., Marette A. (2007). Role of dietary proteins and amino acids in the pathogenesis of insulin resistance. Annu. Rev. Nutr..

[170] Tucker L.A., Erickson A., Lecheminant J.D., Bailey B.W. (2015). Dairy Consumption and Insulin Resistance: The Role of Body Fat, Physical Activity, and Energy Intake. J. Diabetes Res..

[171] Melnik B.C., John S.M., Schmitz G. (2013). Milk is not just food but most likely a genetic transfection system activating mTORC1 signaling for postnatal growth. Nutr. J..

[172] Orgeron M.L., Stone K.P., Wanders D., Cortez C.C., Van N.T., Gettys T.W. (2014). The impact of dietary methionine restriction on biomarkers of metabolic health. Progress in Molecular Biology and Translational Science.

[173] McCarty M.F., Barroso-Aranda J., Contreras F. (2009). The low-methionine content of vegan diets may make methionine restriction feasible as a life extension strategy. Med. Hypotheses.

[174] AsghariHanjani N., Vafa M. (2019). The role of IGF-1 in obesity, cardiovascular disease, and cancer. Med. J. Islam. Repub. Iran.

[175] Le Marchand-Brustel Y., Heydrick S.J., Jullien D., Gautier N., Van Obberghen E. (1995). Effect of insulin and insulin-like growth factor-1 on glucose transport and its transporters in soleus muscle of lean and obese mice. Metabolism.

[176] Gjedsted J., Gormsen L.C., Nielsen S., Schmitz O., Djurhuus C.B., Keiding S., Ørskov H., Tønnesen E., Møller N. (2007). Effects of a 3-day fast on regional lipid and glucose metabolism in human skeletal muscle and adipose tissue. Acta Physiol..

[177] Wurzburger M.I., Prelevic G.M., Sonksen P.H., Balint-Peric L.A., Wheeler M. (1993). The effect of recombinant human growth hormone on regulation of growth hormone secretion and blood glucose in insulin-dependent diabetes. J. Clin. Endocrinol. Metab..

[178] Li R., Ferreira M., Cooke M., La Bounty P., Campbell B., Greenwood M., Willoughby D., Kreider R. (2015). Co-ingestion of carbohydrate with branched-chain amino acids or L-leucine does not preferentially increase serum IGF-1 and expression of myogenic-related genes in response to a single bout of resistance exercise. Amino Acids.

[179] Church D.D., Schwarz N.A., Spillane M.B., McKinley-Barnard S.K., Andre T.L., Ramirez A.J., Willoughby D.S. (2016). l-Leucine Increases Skeletal Muscle IGF-1 but Does Not Differentially Increase Akt/mTORC1 Signaling and Serum IGF-1 Compared to Ursolic Acid in Response to Resistance Exercise in Resistance-Trained Men. J. Am. Coll. Nutr..

[180] Biagetti B., Herance J.R., Ferrer R., Aulinas A., Palomino-Schätzlein M., Mesa J., Castaño J.P., Luque R.M., Simó R. (2019). Metabolic Fingerprint of Acromegaly and its Potential Usefulness in Clinical Practice. J. Clin. Med..

[181] Mastrangelo A., Martos-Moreno G., Rupérez F.J., Chowen J.A., Barbas C., Argente J. (2018). Metabolomics changes in patients with PAPP-A2 deficiency in response to rhIGF1 treatment. Growth Horm. IGF Res..

[182] Sawa R., Nishida H., Yamamoto Y., Wake I., Kai N., Kikkawa U., Okimura Y. (2018). Growth hormone and Insulin-like growth factor-I (IGF-I) modulate the expression of L-type amino acid transporters in the muscles of spontaneous dwarf rats and L6 and C2C12 myocytes. Growth Horm. IGF Res..

[183] Iqbal J., Hussain M.M. (2009). Intestinal lipid absorption. Am. J. Physiol. Endocrinol. Metab..

[184] Quabbe H.J., Bratzke H.J., Siegers U., Elban K. (1972). Studies on the relationship between plasma free fatty acids and growth hormone secretion in man. J. Clin. Investig..

[185] Moller N., Jorgensen J.O.L., Schmitz O., Moller J., Christiansen J.S., Alberti K.G.M.M., Orskov H. (1990). Effects of a growth hormone pulse on total and forearm substrate fluxes in humans. Am. J. Physiol. Endocrinol. Metab..

[186] Neely R.D.G., Rooney D.P., Bell P.M., Bell N.P., Sheridan B., Atkinson A.B., Trimble E.R. (1992). Influence of growth hormone on glucose-glucose 6-phosphate cycle and insulin action in normal humans. Am. J. Physiol. Endocrinol. Metab..

[187] Santomauro A.T.M.G., Boden G., Silva M.E.R., Rocha D.M., Santos R.F., Ursich M.J.M., Strassmann P.G., Wajchenberg B.L. (1999). Overnight lowering of free fatty acids with acipimox improves insulin resistance and glucose tolerance in obese diabetic and nondiabetic subjects. Diabetes.

[188] Zachmann M., Fernandez F., Tassinari D., Thakker R., Prader A. (1980). Anthropometric measurements in patients with growth hormone deficiency before treatment with human growth hormone. Eur. J. Pediatr..

[189] Salomon F., Cuneo R.C., Hesp R., Sönksen P.H. (1989). The Effects of Treatment with Recombinant Human Growth Hormone on Body Composition and Metabolism in Adults with Growth Hormone Deficiency. N. Engl. J. Med..

[190] Davidson M.B. (1987). Effect of growth hormone on carbohydrate and lipid metabolism. Endocr. Rev..

[191] Kreitschmann-Andermahr I., Suarez P., Jennings R., Evers N., Brabant G. (2010). GH/IGF-I regulation in obesity—Mechanisms and practical consequences in children and adults. Horm. Res. Paediatr..

[192] Fuccella L.M., Goidaniga G., Lovisolo P., Maggi E., Musatti L., Mandelli V., Sirtori C.R. (1980). Inhibition of lipolysis by nicotinic acid and by acipimox. Clin. Pharmacol. Ther..

[193] Cordido F., Alvarez-Castro P., Isidro M.L., Casanueva F.F., Dieguez C. (2003). Comparison between insulin tolerance test, growth hormone (GH)-releasing hormone (GHRH), GHRH plus acipimox and GHRH plus GH-releasing peptide-6 for the diagnosis of adult GH deficiency in normal subjects, obese hypopituitary patients. Eur. J. Endocrinol..

[194] Glass A.R., Burman K.D., Dahms W.T., Boehm T.M. (1981). Endocrine function in human obesity. Metabolism.

[195] Peino R., Cordido F., Peñalva A., Alvarez C.V., Dieguez C., Casanueva F.F. (1996). Acipimox-mediated plasma free fatty acid depression per se stimulates growth hormone (GH) secretion in normal subjects and potentiates the response to other GH-releasing stimuli. J. Clin. Endocrinol. Metab..

[196] Imaki T., Shibasaki T., Masuda A., Hotta M., Yamauchi N., Demura H., Shizume K., Wakabayashi I., Ling N. (1986). The effect of glucose and free fatty acids on growth hormone (gh)-releasing factor-mediated gh secretion in rats. Endocrinology.

[197] Casanueva F.F. (1992). Physiology of growth hormone secretion and action. Endocrinol. Metab. Clin. N. Am..

[198] Iranmanesh A., Veldhuis J. (1992). Clinical pathophysiology of the somatotropic (GH) axis in adults. Endocrinol. Metab. Clin. North Am..

[199] Jenkins R.C., Ross R.J.M. (1996). Acquired growth hormone resistance in catabolic states. Baillieres. Clin. Endocrinol. Metab..

[200] Scanlon M.F., Issa B.G., Dieguez C. (1996). Regulation of growth hormone secretion. Horm. Res. Paediatr..

[201] Bolinder J., Lindblad A., Engfeldt P., Arner P. (1987). Studies of acute effects of insulin-like growth factors I and II in human fat cells. J. Clin. Endocrinol. Metab..

[202] Pratipanawatr T., Pratipanawatr W., Rosen C., Berria R., Bajaj M., Cusi K., Mandarino L., Kashyap S., Belfort R., DeFronzo R.A. (2002). Effect of IGF-I on FFA and glucose metabolism in control and type 2 diabetic subjects. Am. J. Physiol. Endocrinol. Metab..

[203] Fang X.L., Shu G., Zhang Z.Q., Wang S.B., Zhu X.T., Gao P., Xi Q.Y., Zhang Y.L., Jiang Q.Y. (2012). Roles of α-linolenic acid on IGF-I secretion and GH/IGF system gene expression in porcine primary hepatocytes. Mol. Biol. Rep..

[204] Yen P.M., Tashjian A.H. (1981). Short chain fatty acids increase prolactin and growth hormone production and alter cell morphology in the GH3 strain of rat pituitary cells. Endocrinology.

[205] Kato S.I., Sato K., Chida H., Roh S.G., Ohwada S., Sato S., Guilloteau P., Katoh K. (2011). Effects of Na-butyrate supplementation in milk formula on plasma concentrations of GH and insulin, and on rumen papilla development in calves. J. Endocrinol..

[206] Ishiwata H., Nagano M., Sasaki Y., Chen C., Katoh K. (2000). Short-chain fatty acids inhibit the release and content of growth hormone in anterior pituitary cells of the goat. Gen. Comp. Endocrinol..

[207] Wang J.F., Fu S.P., Li S.N., Hu Z.M., Xue W.J., Li Z.Q., Huang B.X., Lv Q.K., Liu J.X., Wang W. (2013). Short-chain fatty acids inhibit growth hormone and prolactin gene transcription via cAMP/PKA/CREB signaling pathway in dairy cow anterior pituitary cells. Int. J. Mol. Sci..

[208] Fu S.P., Liu B.R., Wang J.F., Xue W.J., Liu H.M., Zeng Y.L., Huang B.X., Li S.N., Lv Q.K., Wang W. (2015). β-hydroxybutyric acid inhibits growth hormone-releasing hormone synthesis and secretion through the GPR109A/extracellular signal-regulated 1/2 signalling pathway in the hypothalamus. J. Neuroendocrinol..

[209] Pérez-Fernandez R., Alonso M., Segura C., Muñoz I., García-Caballero T., Diéguez C. (1996). Vitamin D receptor gene expression in human pituitary gland. Life Sci..

[210] Giordano M., Godi M., Mellone S., Petri A., Vivenza D., Tiradani L., Carlomagno Y., Ferrante D., Arrigo T., Corneli G. (2008). A functional common polymorphism in the vitamin D-responsive element of the GH1 promoter contributes to isolated growth hormone deficiency. J. Clin. Endocrinol. Metab..

[211] Seoane S., Perez-Fernandez R. (2006). The vitamin D receptor represses transcription of the pituitary transcription factor Pit-1 gene without involvement of the retinoid X receptor. Mol. Endocrinol..

[212] Ameri P., Giusti A., Boschetti M., Murialdo G., Minuto F., Ferone D. (2013). Interactions between vitamin D and IGF-I: From physiology to clinical practice. Clin. Endocrinol. (Oxf.).

[213] Keisala T., Minasyan A., Lou Y.R., Zou J., Kalueff A.V., Pyykkö I., Tuohimaa P. (2009). Premature aging in vitamin D receptor mutant mice. J. Steroid Biochem. Mol. Biol..

[214] Ciresi A., Giordano C. (2017). Vitamin D across growth hormone (GH) disorders: From GH deficiency to GH excess. Growth Horm. IGF Res..

[215] Robson H., Siebler T., Shalet S.M., Williams G.R. (2002). Interactions between GH, IGF-I, glucocorticoids, and thyroid hormones during skeletal growth. Pediatr. Res..

[216] Song Y., Kato S., Fleet J.C. (2003). Vitamin D receptor (VDR) knockout mice reveal VDR-independent regulation of intestinal calcium absorption and ECaC2 and calbindin D9k mRNA. J. Nutr..

[217] Liao L., Chen X., Wang S., Parlow A.F., Xu J. (2008). Steroid Receptor Coactivator 3 Maintains Circulating Insulin-Like Growth Factor I (IGF-I) by Controlling IGF-Binding Protein 3 Expression. Mol. Cell. Biol..

[218] Wei S., Tanaka H., Kubo T., Ono T., Kanzaki S., Seino Y. (1997). Growth hormone increases serum 1,25-dihydroxyvitamin D levels and decreases 24,25-dihydroxyvitamin D levels in children with growth hormone deficiency. Eur. J. Endocrinol..

[219] Halhali A., Díaz L., Sánchez I., Garabédian M., Bourges H., Larrea F. (1999). Effects of IGF-I on 1,25-dihydroxyvitamin D3 synthesis by human placenta in culture. Mol. Hum. Reprod..

[220] Guibourdenche J., Djakouré C., Porquet D., Pagésy P., Rochette-Egly C., Peillon F., Li J.Y., Evain-Brion D. (1997). Retinoic acid stimulates growth hormone synthesis in human somatotropic adenoma cells: Characterization of its nuclear receptors. J. Cell. Biochem..

[221] Prodam F., Caputo M., Mele C., Marzullo P., Aimaretti G. (2021). Insights into non-classic and emerging causes of hypopituitarism. Nat. Rev. Endocrinol..

[222] Djakoure C., Guibourdenche J., Porquet D., Pagesy P., Peillon F., Li J.Y., Evain-Brion D. (1996). Vitamin A and retinoic acid stimulate within minutes cAMP release and growth hormone secretion in human pituitary cells. J. Clin. Endocrinol. Metab..

[223] Murray M., Butler A.M., Fiala-Beer E., Su G.M. (2005). Phospho-STAT5 accumulation in nuclear fractions from vitamin A-deficient rat liver. FEBS Lett..

[224] Raifen R., Altman Y., Zadik Z. (1996). Vitamin A levels and growth hormone axis. Horm. Res. Paediatr..

[225] Evain-Brion D., Fjellestad-Paulsen A., Czernichow P., Porquet D., Thérond P., Grenèche M.O., François L. (1994). Vitamin A deficiency and nocturnal growth hormone secretion in short children. Lancet.

[226] Mohn A., Di Marzio D., Giannini C., Capanna R., Marcovecchio M., Chiarelli F. (2005). Alterations in the oxidant-antioxidant status in prepubertal children with growth hormone deficiency: Effect of growth hormone replacement therapy. Clin. Endocrinol. (Oxf.).

[227] Ren S.G., Melmed S. (2006). Pyridoxal phosphate inhibits pituitary cell proliferation and hormone secretion. Endocrinology.

[228] Báez-Saldaña A., Gutiérrez-Ospina G., Chimal-Monroy J., Fernandez-Mejia C., Saavedra R. (2009). Biotin deficiency in mice is associated with decreased serum availability of insulin-like growth factor-I. Eur. J. Nutr..

[229] Waters M.J., Shang C.A., Behncken S.N., Tam S.P., Li H., Shen B., Lobie P.E. (1999). Growth hormone as a cytokine. Clin. Exp. Pharmacol. Physiol..

[230] Roman-Garcia P., Quiros-Gonzalez I., Mottram L., Lieben L., Sharan K., Wangwiwatsin A., Tubio J., Lewis K., Wilkinson D., Santhanam B. (2014). Vitamin B12-dependent taurine synthesis regulates growth and bone mass. J. Clin. Investig..

[231] Kamenický P., Mazziotti G., Lombès M., Giustina A., Chanson P. (2014). Growth hormone, insulin-like growth factor-1, and the kidney: Pathophysiological and clinical implications. Endocr. Rev..

[232] Feld S., Hirschberg R. (1996). Growth Hormone, the Insulin-Like Growth Factor System, and the Kidney. Endocr. Rev..

[233] McCormick S.D., Bradshaw D. (2006). Hormonal control of salt and water balance in vertebrates. Gen. Comp. Endocrinol..

[234] Rosen T., Bosaeus I., Tolli J., Lindstedt G., Bengtsson B.A. (1993). Increased body fat mass and decreased extracellular fluid volume in adults with growth hormone deficiency. Clin. Endocrinol..

[235] Dimke H., Flyvbjerg A., Frische S. (2007). Acute and chronic effects of growth hormone on renal regulation of electrolyte and water homeostasis. Growth Horm. IGF Res..

[236] Ikkos D., Luft R., Sjogren B. (1954). Body water and sodium in patients with acromegaly. J. Clin. Investig..

[237] Flyvbjerg A., Dørup I., Everts M.E., Ørskov H. (1991). Evidence that potassium deficiency induces growth retardation through reduced circulating levels of growth hormone and insulin-like growth factor I. Metabolism.

[238] Flyvbjerg A., Marshall S.M., Frystyk J., Rasch R., Bornfeldt K.E., Arnqvist H., Jensen P.K., Pallesen G., Orskov H. (1992). Insulin-like growth factor I in initial renal hypertrophy in potassium- depleted rats. Am. J. Physiol. Ren. Fluid Electrolyte Physiol..

[239] Zivadinovic D., Tomić M., Yuan D., Stojilkovic S.S. (2002). Cell-type specific messenger functions of extracellular calcium in the anterior pituitary. Endocrinology.

[240] Nussinovitch I. (2018). Tive supervision enhance. Endocrinology.

[241] Coiro V., Volpi R., Capretti L., Finardi L., Magotti M.G., Manfredi G., Chiodera P., Jotti G.S. (2004). Inhibition of growth hormone secretion in mild primary hyperparathyroidism. Horm. Res..

[242] Romoli R., Lania A., Mantovani G., Corbetta S., Persani L., Spada A. (1999). Expression of Calcium-Sensing Receptor and Characterization of Intracellular Signaling in Human Pituitary Adenomas1. J. Clin. Endocrinol. Metab..

[243] Ikema S., Horikawa R., Nakano M., Yokouchi K., Yamazaki H., Tanaka T., Tanae A. (2000). Growth and metabolic disturbances in a patient with total parenteral nutrition: A case of hypercalciuric hypercalcemia. Endocr. J..

[244] Fleet J.C., Bruns M.E., Hock J.M., Wood R.J. (1994). Growth hormone and parathyroid hormone stimulate intestinal calcium absorption in aged female rats. Endocrinology.

[245] Hoenderop J.G., Müller D., Van Der Kemp A.W., Hartog A., Suzuki M., Ishibashi K., Imai M., Sweep F., Willems P.H., Van Os C.H. (2001). Calcitriol controls the epithelial calcium channel in kidney. J. Am. Soc. Nephrol..

[246] Kamenický P., Blanchard A., Gauci C., Salenave S., Letierce A., Lombès M., Brailly-Tabard S., Azizi M., Prié D., Souberbielle J.C. (2012). Pathophysiology of renal calcium handling in acromegaly: What lies behind hypercalciuria?. J. Clin. Endocrinol. Metab..

[247] Halloran B.P., Spencer E.M. (1988). Dietary phosphorus and 1,25-dihydroxyvitamin d metabolism: Influence of insulin-like growth factor i. Endocrinology.

[248] Harbison M.D., Gertner J.M. (1990). Permissive action of growth hormone on the renal response to dietary phosphorus deprivation. J. Clin. Endocrinol. Metab..

[249] Saggese G., Baroncelli G.I., Federico G., Bertelloni S. (1995). Effects of growth hormone on phosphocalcium homeostasis and bone metabolism. Horm. Res. Paediatr..

[250] Efthymiadou A., Kritikou D., Mantagos S., Chrysis D. (2016). The effect of GH treatment on serum FGF23 and Klotho in GH-deficient children. Eur. J. Endocrinol..

[251] Hirschberg R., Brunori G., Kopple J.D., Guler H.P. (1993). Effects of insulin-like growth factor I on renal function in normal men. Kidney Int..

[252] Giordano M., DeFronzo R.A. (1995). Acute effect of human recombinant insulin-like growth factor I on renal function in humans. Nephron.

[253] Woda C.B., Halaihel N., Wilson P.V., Haramati A., Levi M., Mulroney S.E. (2004). Regulation of renal NaPi-2 expression and tubular phosphate reabsorption by growth hormone in the juvenile rat. Am. J. Physiol. Ren. Physiol..

[254] Shimada T., Kakitani M., Yamazaki Y., Hasegawa H., Takeuchi Y., Fujita T., Fukumoto S., Tomizuka K., Yamashita T. (2004). Targeted ablation of Fgf23 demonstrates an essential physiological role of FGF23 in phosphate and vitamin D metabolism. J. Clin. Investig..

[255] Schmid C., Neidert M.C., Tschopp O., Sze L., Bernays R.L. (2013). Growth hormone and Klotho. J. Endocrinol..

[256] Narayanan N., Lussier B., French M., Moor B., Kraicer J. (1989). Growth hormone-releasing factor-sensitive adenylate cyclase system of purified somatotrophs: Effects of guanine nucleotides, somatostatin, calcium, and magnesium. Endocrinology.

[257] Dørup I., Flyvbjerg A., Everts M.E., Clausen T. (1991). Role of insulin-like growth factor-1 and growth hormone in growth inhibition induced by magnesium and zinc deficiencies. Br. J. Nutr..

[258] Henneman P.H., Forbes A.P., Moldawer M., Dempsey E.F., Carroll E.L. (1960). Effects of human growth hormone in man. J. Clin. Investig..

[259] Mahlbacher K., Sicuro A., Gerber H., Hulter H.N., Krapf R. (1999). Growth hormone corrects acidosis-induced renal nitrogen wasting and renal phosphate depletion and attenuates renal magnesium wasting in humans. Metabolism.

[260] Estívariz C.F., Ziegler T.R. (1997). Nutrition and the Insulin-Like Growth Factor System. Endocrine.

[261] Millward D.J. (2017). Nutrition, infection and stunting: The roles of deficiencies of individual nutrients and foods, and of inflammation, as determinants of reduced linear growth of children. Nutr. Res. Rev..

[262] Cunningham B.C., Mulkerrin M.G., Wells J.A. (1991). Dimerization of human growth hormone by zinc. Science.

[263] Li R., Hui J., Luo G., Hong P., Li L., Yang Y., Zheng X., Lan H. (2019). Zinc ions increase GH signaling ability through regulation of available plasma membrane-localized GHR. J. Cell. Physiol..

[264] Petkovic V., Miletta M.C., Eblé A., Iliev D.I., Binder G., Flück C.E., Mullis P.E. (2013). Effect of zinc binding residues in growth hormone (GH) and altered intracellular zinc content on regulated GH secretion. Endocrinology.

[265] Jacob R.S., Das S., Ghosh S., Anoop A., Jha N.N., Khan T., Singru P., Kumar A., Maji S.K. (2016). Amyloid formation of growth hormone in presence of zinc: Relevance to its storage in secretory granules. Sci. Rep..

[266] Balaz M., Ukropcova B., Kurdiova T., Gajdosechova L., Vlcek M., Janakova Z., Fedeles J., Pura M., Gasperikova D., Smith S.R. (2015). Adipokine zinc-α2-glycoprotein regulated by growth hormone and linked to insulin sensitivity. Obesity.

[267] MacDonald R.S. (2000). The role of zinc in growth and cell proliferation. J. Nutr..

[268] Roth H.P., Kirchgessner M. (1994). Influence of alimentary zinc deficiency on the concentration of growth hormone (GH), insulin-like growth factor I (IGF-I) and insulin in the serum of force-fed rats. Horm. Metab. Res..

[269] Ninh N.X., Thissen J.P., Maiter D., Adam E., Mulumba N., Ketelslegers J.M. (1995). Reduced liver insulin-like growth factor-I gene expression in young zinc-deprived rats is associated with a decrease in liver growth hormone (GH) receptors and serum GH-binding protein. J. Endocrinol..

[270] Hamza R.T., Hamed A.I., Sallam M.T. (2012). Effect of zinc supplementation on growth Hormone Insulin growth factor axis in short Egyptian children with zinc deficiency. Ital. J. Pediatr..

[271] Ekbote V., Khadilkar A., Chiplonkar S., Mughal Z., Khadilkar V. (2013). Enhanced effect of zinc and calcium supplementation on bone status in growth hormone-deficient children treated with growth hormone: A pilot randomized controlled trial. Endocrine.

[272] de Medeiros Rocha É.D., de Brito N.J.N., Dantas M.M.G., de Araújo Silva A., das Graças Almeida M., Brandão-Neto J. (2015). Effect of Zinc Supplementation on GH, IGF1, IGFBP3, OCN, and ALP in Non-Zinc-Deficient Children. J. Am. Coll. Nutr..

[273] Prodam F., Aimaretti G. (2013). Could zinc supplementation improve bone status in growth hormone (GH) deficient children?. Endocrine.

[274] Bouglé D., Laroche D., Bureau F. (2000). Zinc and iron status and growth in healthy infants. Eur. J. Clin. Nutr..

[275] Stred S.E., Messina J.L. (2003). Identification of hemopexin as a GH-regulated gene. Mol. Cell. Endocrinol..

[276] Weinzimer S.A., Gibson T.B., Collett-Solberg P.F., Khare A., Liu B., Cohen P. (2001). Transferrin Is an Insulin-Like Growth Factor-Binding Protein-3 Binding Protein1. J. Clin. Endocrinol. Metab..

[277] Miyagawa S.I., Kobayashi M., Konishi N., Sato T., Ueda K. (2000). Insulin and insulin-like growth factor I support the proliferation of erythroid progenitor cells in bone marrow through the sharing of receptors. Br. J. Haematol..

[278] Choi J., Kim S. (2004). Association of serum insulin-like growth factor-I and erythropoiesis in relation to body iron status. Ann. Clin. Lab. Sci..

[279] De Vita F., Maggio M., Lauretani F., Crucitti L., Bandinelli S., Mammarella F., Landi F., Ferrucci L., Ceda G.P. (2015). Insulin-like growth factor-1 and anemia in older subjects: The inchianti study. Endocr. Pract..

[280] Vihervuori E., Cook J.D., Siimes M.A. (1997). Iron status of children with short stature during accelerated growth due to growth hormone treatment. Acta Paediatr. Int. J. Paediatr..

[281] Anwar U., ZR A., Filteau S., Sullivan K., Tomkins A. (1997). The impact of maternal supplementation with a single dose of oral iodized poppyseed oil on infant thyroid status in rural Bangladesh. Trans. R. Soc. Trop. Med. Hyg..

[282] Zimmermann M.B., Jooste P.L., Mabapa N.S., Mbhenyane X., Schoeman S., Biebinger R., Chaouki N., Bozo M., Grimci L., Bridson J. (2007). Treatment of iodine deficiency in school-age children increases insulin-like growth factor (IGF)-I and IGF binding protein-3 concentrations and improves somatic growth. J. Clin. Endocrinol. Metab..

[283] Crew M., Spindler S. (1986). Thyroid hormone regulation of the transfected rat growth hormone promoter. J. Biol. Chem..

[284] Miell J.P., Taylor A.M., Zini M., Maheshwari H.G., Ross R.J.M., Valcavi R. (1993). Effects of hypothyroidism and hyperthyroidism on insulin-like growth factors (IGFs) and growth hormone- and IGF-binding proteins. J. Clin. Endocrinol. Metab..

[285] Nanto-Salonen K., Muller H.L., Hoffman A.R., Vu T.H., Rosenfeld R.G. (1993). Mechanisms of thyroid hormone action on the insulinlike growth factor system: All thyroid hormone effects are not growth hormone mediated. Endocrinology.

[286] Thorlacius-Ussing O., Flyvbjerg A., Esmann J. (1987). Evidence that selenium induces growth retardation through reduced growth hormone and somatomedin c production. Endocrinology.

[287] Rayman M.P. (2000). The importance of selenium to human health. Lancet.

[288] Ren G., Ali T., Chen W., Han D., Zhang L., Gu X., Zhang S., Ding L., Fanning S., Han B. (2016). The role of selenium in insulin-like growth factor I receptor (IGF-IR) expression and regulation of apoptosis in mouse osteoblasts. Chemosphere.

[289] Wang J., Lian S., He X., Yu D., Liang J., Sun D., Wu R. (2018). Selenium deficiency induces splenic growth retardation by deactivating the IGF-1R/PI3K/Akt/mTOR pathway. Metallomics.

[290] Moreno-Reyes R., Egrise D., Nève J., Pasteels J.L., Schoutens A. (2001). Selenium deficiency-induced growth retardation is associated with an impaired bone metabolism and osteopenia. J. Bone Miner. Res..

[291] Maggio M., De Vita F.D., Lauretani F., Buttò V., Bondi G., Cattabiani C., Nouvenne A., Meschi T., Dall’Agli E., Ceda G.P. (2013). IGF-1, the cross road of the nutritional, inflammatory and hormonal pathways to frailty. Nutrients.

[292] Aydin K., Bideci A., Kendirci M., Cinaz P., Kurtoglu S. (2002). Insulin-like growth factor-I and insulin-like growth factor binding protein-3 levels of children living in an iodine- and selenium-deficient endemic goiter area. Biol. Trace Elem. Res..

[293] Maggio M., Ceda G.P., Lauretani F., Bandinelli S., Dall’Aglio E., Guralnik J.M., Paolisso G., Semba R.D., Nouvenne A., Borghi L. (2010). Association of plasma selenium concentrations with total IGF-1 among older community-dwelling adults: The InCHIANTI study. Clin. Nutr..

[294] Young N.J., Metcalfe C., Gunnell D., Rowlands M.A., Lane J.A., Gilbert R., Avery K.N.L., Davis M., Neal D.E., Hamdy F.C. (2012). A cross-sectional analysis of the association between diet and insulin-like growth factor (IGF)-I, IGF-II, IGF-binding protein (IGFBP)-2, and IGFBP-3 in men in the United Kingdom. Cancer Causes Control.

[295] Janjuha R., Bunn D., Hayhoe R., Hooper L., Abdelhamid A., Mahmood S., Hayden-Case J., Appleyard W., Morris S., Welch A. (2020). Effects of dietary or supplementary micronutrients on sex hormones and IGF-1 in middle and older age: A systematic review and meta-analysis. Nutrients.

[296] Werner L., Korc M., Brannon P.M. (1987). Effects of manganese deficiency and dietary composition on rat pancreatic enzyme content. J. Nutr..

[297] Clegg M.S., Donovan S.M., Monaco M.H., Baly D.L., Ensunsa J.L., Keen C.L. (1998). The influence of manganese deficiency on serum IGF-1 and IGF binding proteins in the male rat. Proc. Soc. Exp. Biol. Med..

[298] Bryan M.R., Nordham K.D., Rose D.I.R., O’Brien M.T., Joshi P., Foshage A.M., Gonçalves F.M., Nitin R., Uhouse M.A., Aschner M. (2020). Manganese Acts upon Insulin/IGF Receptors to Phosphorylate AKT and Increase Glucose Uptake in Huntington’s Disease Cells. Mol. Neurobiol..

[299] Hiney J.K., Srivastava V.K., Dees W. (2011). Les Manganese induces IGF-1 and cyclooxygenase-2 gene expressions in the basal hypothalamus during prepubertal female development. Toxicol. Sci..

[300] Yang W., Wang J., Zhu X., Gao Y., Liu Z., Zhang L., Chen H., Shi X., Yang L., Liu G. (2012). High lever dietary copper promote ghrelin gene expression in the fundic gland of growing pigs. Biol. Trace Elem. Res..

[301] Roughead Z.K.F., Lukaski H.C. (2003). Inadequate copper intake reduces serum insulin-like growth factor-I and bone strength in growing rats fed graded amounts of copper and zinc. J. Nutr..

[302] Yang W., Wang J., Liu L., Zhu X., Wang X., Liu Z., Wang Z., Yang L., Liu G. (2011). Effect of high dietary copper on somatostatin and growth hormone-releasing hormone levels in the hypothalami of growing pigs. Biol. Trace Elem. Res..

[303] Wang M.Q., Xu Z.R., Li W.F., Jiang Z.G. (2009). Effect of chromium nanocomposite supplementation on growth hormone pulsatile secretion and mRNA expression in finishing pigs. J. Anim. Physiol. Anim. Nutr..

[304] Merimee T.J., Pulkkinen A.J., Burton C.E. (1976). Diet-induced alterations of hgh secretion in man. J. Clin. Endocrinol. Metab..

[305] Harber M.P., Schenk S., Barkan A.L., Horowitz J.F. (2005). Effects of dietary carbohydrate restriction with high protein intake on protein metabolism and the somatotropic axis. J. Clin. Endocrinol. Metab..

[306] McCargar L.J., Clandinin M.T., Belcastro A.N., Walker K. (1989). Dietary carbohydrate-to-fat ratio: Influence on whole-body nitrogen retention, substrate utilization, and hormone response in healthy male subjects. Am. J. Clin. Nutr..

[307] Darling-Raedeke M., Thornton W.H., MacDonald R.S. (1998). Growth hormone and igf-i plasma concentrations and macronutrient intake measured in a free-living elderly population during a one-year period. J. Am. Coll. Nutr..

[308] Fontana L., Partridge L., Longo V.D. (2010). Extending healthy life span-from yeast to humans. Science.

[309] López-Otín C., Blasco M.A., Partridge L., Serrano M., Kroemer G. (2013). The hallmarks of aging. Cell.

[310] Holloszy J.O., Fontana L. (2007). Caloric restriction in humans. Exp. Gerontol..

[311] Efeyan A., Comb W.C., Sabatini D.M. (2015). Nutrient-sensing mechanisms and pathways. Nature.

[312] Redman L.M., Smith S.R., Burton J.H., Martin C.K., Il’yasova D., Ravussin E. (2018). Metabolic Slowing and Reduced Oxidative Damage with Sustained Caloric Restriction Support the Rate of Living and Oxidative Damage Theories of Aging. Cell Metab..

[313] Fontana L., Klein S. (2007). Aging, adiposity, and calorie restriction. J. Am. Med. Assoc..

[314] Mercken E.M., Crosby S.D., Lamming D.W., Jebailey L., Krzysik-Walker S., Villareal D.T., Capri M., Franceschi C., Zhang Y., Becker K. (2013). Calorie restriction in humans inhibits the PI3K/AKT pathway and induces a younger transcription profile. Aging Cell.

[315] Milman S., Atzmon G., Huffman D.M., Wan J., Crandall J.P., Cohen P., Barzilai N. (2014). Low insulin-like growth factor-1 level predicts survival in humans with exceptional longevity. Aging Cell.

[316] Renehan A.G., Zwahlen M., Minder C., O’Dwyer S.T., Shalet S.M., Egger M. (2004). Insulin-like growth factor (IGF)-I, IGF binding protein-3, and cancer risk: Systematic review and meta-regression analysis. Lancet.

[317] Bartke A. (2011). Pleiotropic effects of growth hormone signaling in aging. Trends Endocrinol. Metab..

[318] Al-Regaiey K.A., Masternak M.M., Bonkowski M., Sun L., Bartke A. (2005). Long-lived growth hormone receptor knockout mice: Interaction of reduced insulin-like growth factor I/insulin signaling and caloric restriction. Endocrinology.

[319] Bartke A. (2005). Role of the growth hormone/insulin-like growth factor system in mammalian aging. Endocrinology.

[320] Longo V.D., Fontana L. (2010). Calorie restriction and cancer prevention: Metabolic and molecular mechanisms. Trends Pharmacol. Sci..

[321] Sabatino F., Masoro E.J., McMahan C.A., Kuhn R.W. (1991). Assessment of the role of the glucocorticoid system in aging processes and in the action of food restriction. J. Gerontol..

[322] Redman L.M., Veldhuis J.D., Rood J., Smith S.R., Williamson D., Ravussin E. (2010). The effect of caloric restriction interventions on growth hormone secretion in nonobese men and women. Aging Cell.

[323] Cohen D.E., Supinski A.M., Bonkowski M.S., Donmez G., Guarente L.P. (2009). Neuronal SIRT1 regulates endocrine and behavioral responses to calorie restriction. Genes Dev..

[324] Sonntag W.E., Xu X., Ingram R.L., D’Costa A. (1995). Moderate caloric restriction alters the subcellular distribution of somatostatin mRNA and increases growth hormone pulse amplitude in aged animals. Neuroendocrinology.

[325] Caputo M., Mele C., Ferrero A., Leone I., Daffara T., Marzullo P., Prodam F., Aimaretti G. (2021). Dynamic tests in pituitary endocrinology: Pitfalls in interpretation during aging. Neuroendocrinology.

[326] Bartke A., Wright J.C., Mattison J.A., Ingram D.K., Miller R.A., Roth G.S. (2001). Extending the lifespan of long-lived mice. Nature.

[327] Dani S.U., Dani M.A.C., Freire I.L., Gouvea S.P., Knackfuss F.B., Lima F.P., Mercadante M.E.Z., Monteiro E., Paggiaro S.M.G., Razook A.G. (2010). Survival of the thriftiest: Restricted nurture reveals the thrifty nature of a growth gene in Bos indicus. Genet. Mol. Res..

[328] Sonntag W.E., Csiszar A., De Cabo R., Ferrucci L., Ungvari Z. (2012). Diverse roles of growth hormone and insulin-like growth factor-1 in mammalian aging: Progress and controversies. J. Gerontol. Ser. A Biol. Sci. Med. Sci..

[329] Suh Y., Atzmon G., Cho M.O., Hwang D., Liu B., Leahy D.J., Barzilai N., Cohen P. (2008). Functionally significant insulin-like growth factor I receptor mutations in centenarians. Proc. Natl. Acad. Sci. USA.

[330] Guevara-Aguirre J., Balasubramanian P., Guevara-Aguirre M., Wei M., Madia F., Cheng C.W., Hwang D., Martin-Montalvo A., Saavedra J., Ingles S. (2011). Growth hormone receptor deficiency is associated with a major reduction in pro-aging signaling, cancer, and diabetes in humans. Sci. Transl. Med..

[331] Fontana L., Weiss E.P., Villareal D.T., Klein S., Holloszy J.O. (2008). Long-term effects of calorie or protein restriction on serum IGF-1 and IGFBP-3 concentration in humans. Aging Cell.

[332] Gavrilova N.S., Gavrilov L.A. (2012). Comments on dietary restriction, okinawa diet and longevity. Gerontology.

[333] Willcox D.C., Willcox B.J., Todoriki H., Curb J.D., Suzuki M. (2006). Caloric restriction and human longevity: What can we learn from the Okinawans?. Biogerontology.

[334] Willcox D., Willcox B., Yasura S., Ashitomi I., Suzuki M. (2012). Gender gap in healthspan and life expectancy in Okinawa: Health behaviours. Asian J. Gerontol. Geriatr..

[335] Lynch C.J., Adams S.H. (2014). Branched-chain amino acids in metabolic signalling and insulin resistance. Nat. Rev. Endocrinol..

[336] Catenacci V.A., Pan Z., Ostendorf D., Brannon S., Gozansky W.S., Mattson M.P., Martin B., MacLean P.S., Melanson E.L., Troy Donahoo W. (2016). A randomized pilot study comparing zero-calorie alternate-day fasting to daily caloric restriction in adults with obesity. Obesity.

[337] Varady K.A., Bhutani S., Church E.C., Klempel M.C. (2009). Short-term modified alternate-day fasting: A novel dietary strategy for weight loss and cardioprotection in obese adults. Am. J. Clin. Nutr..

[338] Harvie M., Wright C., Pegington M., McMullan D., Mitchell E., Martin B., Cutler R.G., Evans G., Whiteside S., Maudsley S. (2013). The effect of intermittent energy and carbohydrate restriction v. daily energy restriction on weight loss and metabolic disease risk markers in overweight women. Br. J. Nutr..

[339] Varady K.A., Bhutani S., Klempel M.C., Kroeger C.M., Trepanowski J.F., Haus J.M., Hoddy K.K., Calvo Y. (2013). Alternate day fasting for weight loss in normal weight and overweight subjects: A randomized controlled trial. Nutr. J..

[340] Varady K.A., Roohk D.J., Loe Y.C., McEvoy-Hein B.K., Hellerstein M.K. (2007). Effects of modified alternate-day fasting regimens on adipocyte size, triglyceride metabolism, and plasma adiponectin levels in mice. J. Lipid Res..

[341] Varady K.A., Allister C.A., Roohk D.J., Hellerstein M.K. (2010). Improvements in body fat distribution and circulating adiponectin by alternate-day fasting versus calorie restriction. J. Nutr. Biochem..

[342] Varady K.A., Hudak C.S., Hellerstein M.K. (2009). Modified alternate-day fasting and cardioprotection: Relation to adipose tissue dynamics and dietary fat intake. Metabolism..

[343] Ash S., Reeves M.M., Yeo S., Morrison G., Carey D., Capra S. (2003). Effect of intensive dietetic interventions on weight and glycaemic control in overweight men with Type II diabetes: A randomised trial. Int. J. Obes..

[344] Klempel M.C., Kroeger C.M., Bhutani S., Trepanowski J.F., Varady K.A. (2012). Intermittent fasting combined with calorie restriction is effective for weight loss and cardio-protection in obese women. Nutr. J..

[345] Hoddy K.K., Kroeger C.M., Trepanowski J.F., Barnosky A., Bhutani S., Varady K.A. (2014). Meal timing during alternate day fasting: Impact on body weight and cardiovascular disease risk in obese adults. Obesity.

[346] Eshghinia S., Mohammadzadeh F. (2013). The effects of modified alternate-day fasting diet on weight loss and CAD risk factors in overweight and obese women. J. Diabetes Metab. Disord..

[347] Zuo L., He F., Tinsley G.M., Pannell B.K., Ward E., Arciero P.J. (2016). Comparison of high-protein, intermittent fasting low-calorie diet and heart healthy diet for vascular health of the obese. Front. Physiol..

[348] Harvie M.N., Sims A.H., Pegington M., Spence K., Mitchell A., Vaughan A.A., Allwood J.W., Xu Y., Rattray N.J.W., Goodacre R. (2016). Intermittent energy restriction induces changes in breast gene expression and systemic metabolism. Breast Cancer Res..

[349] Wegman M.P., Guo M.H., Bennion D.M., Shankar M.N., Chrzanowski S.M., Goldberg L.A., Xu J., Williams T.A., Lu X., Hsu S.I. (2015). Practicality of Intermittent Fasting in Humans and its Effect on Oxidative Stress and Genes Related to Aging and Metabolism. Rejuvenation Res..

[350] Ułamek-Kozioł M., Czuczwar S.J., Pluta R., Januszewski S. (2019). Ketogenic diet and epilepsy. Nutrients.

[351] Caprio M., Infante M., Moriconi E., Armani A., Fabbri A., Mantovani G., Mariani S., Lubrano C., Poggiogalle E., Migliaccio S. (2019). Very-low-calorie ketogenic diet (VLCKD) in the management of metabolic diseases: Systematic review and consensus statement from the Italian Society of Endocrinology (SIE). J. Endocrinol. Investig..

[352] Coopmans E.C., Berk K.A.C., El-Sayed N., Neggers S.J.C.M.M., van der Lely A.J. (2020). Eucaloric Very-Low-Carbohydrate Ketogenic Diet in Acromegaly Treatment. N. Engl. J. Med..

[353] Kossoff E.H., Zupec-Kania B.A., Auvin S., Ballaban-Gil K.R., Christina Bergqvist A.G., Blackford R., Buchhalter J.R., Caraballo R.H., Cross J.H., Dahlin M.G. (2018). Optimal clinical management of children receiving dietary therapies for epilepsy: Updated recommendations of the International Ketogenic Diet Study Group. Epilepsia Open.

[354] Peterson S.J., Tangney C.C., Pimentel-Zablah E.M., Hjelmgren B., Booth G., Berry-Kravis E. (2005). Changes in growth and seizure reduction in children on the ketogenic diet as a treatment for intractable epilepsy. J. Am. Diet. Assoc..

[355] Spulber G., Spulber S., Hagenäs L., Åmark P., Dahlin M. (2009). Growth dependence on insulin-like growth factor-1 during the ketogenic diet. Epilepsia.

[356] Vining E.P.G., Pyzik P., McGrogan J., Hladky H., Anand A., Kriegler S., Freeman J.M. (2002). Growth of children on the ketogenic diet. Dev. Med. Child Neurol..

[357] Wiĺliams S., Basualdo-Hammond C., Curtis R., Schuller R. (2002). Growth retardation in children with epilepsy on the ketogenic diet: A retrospective chart review. J. Am. Diet. Assoc..

[358] Groleau V., Schall J.I., Stallings V.A., Bergqvist C.A. (2014). Long-term impact of the ketogenic diet on growth and resting energy expenditure in children with intractable epilepsy. Dev. Med. Child Neurol..

[359] Kim J.T., Kang H.C., Song J.E., Lee M.J., Lee Y.J., Lee E.J., Lee J.S., Kim H.D. (2013). Catch-up growth after long-term implementation and weaning from ketogenic diet in pediatric epileptic patients. Clin. Nutr..

[360] Marchiò M., Roli L., Lucchi C., Costa A.M., Borghi M., Iughetti L., Trenti T., Guerra A., Biagini G. (2019). Ghrelin Plasma Levels After 1 Year of Ketogenic Diet in Children With Refractory Epilepsy. Front. Nutr..

[361] Wibisono C., Rowe N., Beavis E., Kepreotes H., Mackie F.E., Lawson J.A., Cardamone M. (2015). Ten-year single-center experience of the ketogenic diet: Factors influencing efficacy, tolerability, and compliance. J. Pediatr..

[362] Lambrechts D.A.J.E., de Kinderen R.J.A., Vles H.S.H., de Louw A.J., Aldenkamp A.P., Majoie M.J.M. (2015). The MCT-ketogenic diet as a treatment option in refractory childhood epilepsy: A prospective study with 2-year follow-up. Epilepsy Behav..

[363] Dressler A., Stöcklin B., Reithofer E., Benninger F., Freilinger M., Hauser E., Reiter-Fink E., Seidl R., Trimmel-Schwahofer P., Feucht M. (2010). Long-term outcome and tolerability of the ketogenic diet in drug-resistant childhood epilepsy-the austrian experience. Seizure.

[364] Ferraris C., Guglielmetti M., Pasca L., De Giorgis V., Ferraro O.E., Brambilla I., Leone A., De Amicis R., Bertoli S., Veggiotti P. (2019). Impact of the ketogenic diet on linear growth in children: A single-center retrospective analysis of 34 cases. Nutrients.

[365] Armeno M., Verini A., del Pino M., Araujo M.B., Mestre G., Reyes G., Caraballo R.H. (2019). A prospective study on changes in nutritional status and growth following two years of ketogenic diet (KD) therapy in children with refractory epilepsy. Nutrients.

[366] Nation J., Humphrey M., Mackay M., Boneh A. (2014). Linear growth of children on a ketogenic diet: Does the protein-to-energy ratio matter?. J. Child Neurol..

[367] Svedlund A., Hallböök T., Magnusson P., Dahlgren J., Swolin-Eide D. (2019). Prospective study of growth and bone mass in Swedish children treated with the modified Atkins diet. Eur. J. Paediatr. Neurol..

[368] Salas-Salvadó J., Fernández-Ballart J., Ros E., Martínez-González M.A., Fitó M., Estruch R., Corella D., Fiol M., Gómez-Gracia E., Arós F. (2008). Effect of a Mediterranean diet supplemented with nuts on metabolic syndrome status: One-year results of the PREDIMED randomized trial. Arch. Intern. Med..

[369] Muscogiuri G., Barrea L., Laudisio D., Di Somma C., Pugliese G., Salzano C., Colao A., Savastano S. (2019). Somatotropic axis and obesity: Is there any role for the Mediterranean diet?. Nutrients.

[370] Vila G., Jørgensen J.O.L., Luger A., Stalla G.K. (2019). Insulin resistance in patients with acromegaly. Front. Endocrinol..

[371] Jørgensen J.O.L. (2001). Treatment guidelines for acromegaly. Report from a Scandinavian workshop. Growth Horm. IGF Res..

[372] Widmer R.J., Flammer A.J., Lerman L.O., Lerman A. (2015). The Mediterranean diet, its components, and cardiovascular disease. Am. J. Med..

[373] Whitehead T., Robinson D., Allaway S., Syms J., Hale A. (1995). Effect of red wine ingestion on the antioxidant capacity of serum. Clin. Chem..

[374] Lasheras C., Gonzalez S., Huerta J.M., Lombardia C., Ibañez R., Patterson A.M., Fernandez S. (2003). Food habits are associated with lipid peroxidation in an elderly population. J. Am. Diet. Assoc..

[375] Antonini F.M., Petruzzi E., Pinzani P., Orlando C., Petruzzi I., Pazzagli M., Masotti G. (2005). Effect of diet and red wine consumption on serum total antioxidant capacity (TAC), dehydroepiandrosterone-sulphate (DHEAS) and insulin-like growth factor-1 (IGF-1) in Italian centenarians. Arch. Gerontol. Geriatr..

[376] Sambrook P.N., Chen J.S., March L.M., Cameron I.D., Cumming R.G., Lord S.R., Zochling J., Sitoh Y.Y., Lau T.C., Schwarz J. (2004). Serum parathyroid hormone predicts time to fall independent of vitamin D status in a frail elderly population. J. Clin. Endocrinol. Metab..

[377] Bassaganya-Riera J., Berry E.M., Blaak E.E., Burlingame B., le Coutre J., van Eden W., El-Sohemy A., German J.B., Knorr D., Lacroix C. (2021). Goals in Nutrition Science 2020–2025. Front. Nutr..

[378] Solon-Biet S.M., Mitchell S.J., de Cabo R., Raubenheimer D., Le Couteur D.G., Simpson S.J. (2015). Macronutrients and caloric intake in health and longevity. J. Endocrinol..

[379] Johnson S.C. (2018). Nutrient sensing, signaling and ageing: The role of IGF-1 and mTOR in ageing and age-related disease. Subcellular Biochemistry.

[380] Brown-Borg H.M. (2015). The somatotropic axis and longevity in mice. Am. J. Physiol. Endocrinol. Metab..

[381] Simpson S.J., Raubenheimer D. (2007). Caloric restriction and aging revisited: The need for a geometric analysis of the nutritional bases of aging. J. Gerontol. Ser. A Biol. Sci. Med. Sci..

[382] Simpson S., Raubenheimer D. (2012). The Nature of Nutrition. A Unifying Framework Form Animal Adaption to Human Obesity.

